# Equivalent Modeling and Calculation of the Infrared Characteristics of Combined Multi-Regular Triangular Pyramid Cavity Structures Based on Cavity Radiation Theory

**DOI:** 10.3390/s26175457

**Published:** 2026-08-28

**Authors:** Shucheng Zhou, Shengliang Hu, Hai Wu, Yasong Luo

**Affiliations:** Naval University of Engineering, Wuhan 430030, China; m24182601@nue.edu.cn (S.Z.); 0909061010@nue.edu.cn (S.H.); 1109061006@nue.edu.cn (Y.L.)

**Keywords:** cavity radiation theory, regular triangular pyramid cavity, multi-cavity composite structure, effective emissivity, infrared radiation characteristics

## Abstract

To address the high computational complexity of evaluating the infrared radiation characteristics of combined multi-regular triangular pyramid cavity structures and the low efficiency of direct numerical simulation, this paper proposes an equivalent modeling and computational method for infrared characteristics based on cavity radiation theory. Taking a regular icosahedral corner reflector as the object of study, the complex multi-cavity structure is first decomposed into several regular triangular pyramid cavity units. Based on cavity radiation theory, first- and second-order approximate models for the effective emissivity of the opening of a single regular triangular pyramid cavity are derived, and the average effective emissivity is then determined in conjunction with geometric relationships. Subsequently, using the calculated effective emissivity of a single cavity, the multi-cavity composite structure is radiatively equivalent to a regular solid with the same outer contour and uniform surface emissivity, thereby enabling rapid calculation of the infrared radiation characteristics of the complex target. An observation network comprising 82 viewpoints was constructed, and simulation-based validation was conducted under varying temperature, size, and material-emissivity conditions. Based on the data analysis, an empirical correction term was subsequently introduced to compensate for the errors. The results show that, within the emissivity range of 0.2–0.9, the average error between the corrected equivalent model and the simulation results of the original complex structure can be controlled within 1%. Moreover, the model exhibits good stability with respect to variations in target temperature and geometric size. While maintaining computational accuracy, the proposed method improves computational efficiency by approximately 3.8 times compared with direct simulation. The study demonstrates that the proposed method can effectively preserve the main infrared radiation characteristics of combined multi-regular triangular pyramid cavity structures and can provide methodological support for the rapid evaluation and engineering design of the infrared characteristics of such complex cavity targets.

## 1. Introduction

Infrared radiation is the electromagnetic energy spontaneously emitted by all objects in nature with temperatures above absolute zero [1], and it serves as one of the core carriers of information in modern passive detection [2,3], remote sensing imaging [4,5], and thermal radiation measurement systems [6,7]. In recent years, infrared detection and sensing technologies have advanced significantly, as evidenced by the emergence of long-wave infrared detectors [8] and miniaturized mid-wave infrared spectrometers [9]. Meanwhile, infrared technology has also been extended to emerging fields such as biomedical microrobotics [10].

In engineering practice, many targets and devices are not composed of a single smooth outer surface; instead, they contain cavities, aperture arrays, and occluding structures with different scales and aspect ratios. Typical examples include blackbody cavities for radiation calibration [11,12,13], baffles and internal cavity structures used to suppress stray light in opto-mechanical systems [14], perforated or honeycomb thermal control components [15], corner reflectors [16], and various complex composite cavity structures assembled from polyhedral units. The apparent infrared radiation characteristics of such structures often deviate significantly from those directly characterized by the intrinsic emissivity of the material. Their radiation intensity, angular distribution, and even the equivalent emissivity within a given spectral band are strongly modulated by cavity geometry, multiple reflections, and self-occlusion effects [17,18]. Therefore, developing an infrared radiation modeling method for multi-cavity composite structures that possesses both physical interpretability and engineering applicability is of great significance for infrared characteristic evaluation, device design optimization, and system-level simulation.

From a mechanistic perspective, the reason why cavity structures can significantly alter infrared radiation output is that the radiative energy within the cavity undergoes multiple reflections between the inner walls and is gradually absorbed [19,20]. As a result, the cavity aperture exhibits an “effective emissivity” that is higher than the intrinsic emissivity of the material, while also producing angular-dependent radiation emission characteristics [21]. Classical cavity radiation theory provides a fundamental framework for deriving the effective emissivity characteristics of such structures, while most existing studies have focused on the design, calculation, and measurement of high-emissivity blackbody sources [17,22,23,24]. Meanwhile, recent studies in optics and photonics have enhanced light–matter interactions through the design of complex cavity structures. Representative examples include an annular multipass absorption cell based on an off-axis model [25], a photonic Dirac-vortex cavity with spin–orbit angular momentum locking [26], and a hybrid cavity featuring tunable coupling between anapole and Fabry–Pérot resonances [27]. These advances demonstrate the growing interest in the design of complex cavity structures within the optical community. However, systematic engineering-oriented methods for the analytical modeling and rapid calculation of the effective thermal-radiation emissivity of multi-cavity composite targets in the infrared regime remain lacking.

In numerical simulation, Monte Carlo ray tracing (MCRT) and its backward or bidirectional variants, such as the reverse Monte Carlo method (RMCM), have become important tools for infrared radiative transfer analysis because of their ability to handle complex geometries, multiple reflections, and occlusion effects [28,29]. In recent years, various acceleration and coupling frameworks have been proposed to address issues such as convergence efficiency, mesh adaptability, and multiphysics coupling. Examples include unified conduction–convection–radiation frameworks that reduce the cost of stepwise iteration [30], reverse sampling and iterative optimization strategies that improve convergence speed [28], and methods combining unstructured meshes with bidirectional tracing to accommodate complex boundaries [29]. In addition, methods for characterizing complex structures and analyzing the associated data continue to evolve. Examples include three-dimensional cross-scale characterization of curved diffractive microstructures [31] and numerical simulations of active galactic nucleus light curves [32], both of which highlight the challenges involved in addressing complex radiative and geometrical problems. Nevertheless, these methods share a common limitation: when the object under investigation contains a large number of cavity units and requires iterative design and error evaluation under multiple operating conditions, the computational cost may remain a critical barrier to engineering applications. Therefore, there is an urgent need for an equivalent modeling approach that can effectively reduce the computational complexity of evaluating the infrared radiative characteristics of multi-cavity composite structures while preserving the dominant physical mechanisms.

Corner reflectors are typical targets with multi-triangular-pyramid cavity composite structures. As a classical trihedral right-angle reflecting structure, a corner reflector is capable of returning electromagnetic waves incident from a given direction approximately back along the original path in the radar band, thereby generating strong scattering in that direction. For this reason, it has long been widely used in maritime rescue, radar system calibration, and electronic countermeasure applications [33,34,35]. Its interior is formed by the combination of multiple corner-cavity units, where significant multiple reflection and self-occlusion effects exist, which may cause the apparent radiation characteristics in the infrared band to differ markedly from the intrinsic emissivity of the material. Existing studies on corner reflectors have mainly focused on radar and electromagnetic scattering [36,37,38,39,40], whereas publicly available research on radiative transfer modeling, equivalent emissivity characterization, and multi-view radiation characteristic prediction in the infrared band remains relatively limited. There is therefore a pressing need to develop a modeling method that balances computational efficiency and accuracy and can be used for engineering parametric sweeps and design iteration.

Based on the above considerations, this study takes corner reflectors as the primary research object and proposes an equivalent modeling and computational method for their infrared characteristics based on cavity radiation theory. Specifically, the complex multicavity structure is first decomposed into a number of regular triangular-pyramidal cavity units. The effective emissivity of an individual cavity aperture is then derived using cavity radiation theory, and the average effective emissivity is determined in combination with the relevant geometrical relationships. Subsequently, the multicavity composite structure is radiatively represented by a regular solid with the same external profile and a uniform surface emissivity, thereby enabling rapid calculation of its infrared radiative characteristics. Finally, a multi-viewpoint omnidirectional observation network is established, and simulation-based validation is performed using the RMCM. The associated error patterns are analyzed, and correction strategies are proposed. This work provides reusable theoretical and methodological support for the characterization and engineering design of such multicavity composite structures in infrared detection scenarios. Compared with existing studies on cavity radiation, the innovations of this work can be summarized in three aspects. First, it extends the applicability of classical theories. Classical cavity radiation theories, such as the Gouffé and De Vos theories, have primarily been applied to small-aperture cavities, particularly those with rotationally symmetric geometries. In contrast, this study proposes a method for calculating the radiative characteristics of regular triangular-pyramidal cavities with an open base, thereby addressing the lack of analytical modeling approaches for large-aperture cavities with non-axisymmetric geometries. Second, it further improves computational efficiency beyond existing methods. Common numerical approaches for simulating infrared characteristics, such as the Monte Carlo method, generally face a trade-off between accuracy and efficiency when dealing with complex multicavity structures. By developing an equivalent modeling method, this study addresses the problem at the modeling level and achieves efficient computational simulation while maintaining the required accuracy. Third, it broadens the scope of research on the characteristics of corner-reflector targets. To the best of our knowledge, this study is the first to quantitatively calculate the infrared characteristics of corner reflectors, thereby addressing a gap in infrared-related research and providing theoretical support for their subsequent applications in diverse fields.

## 2. Equivalent Calculation Method for the Infrared Characteristics of an Icosahedral Corner Reflector

**Figure 1:**
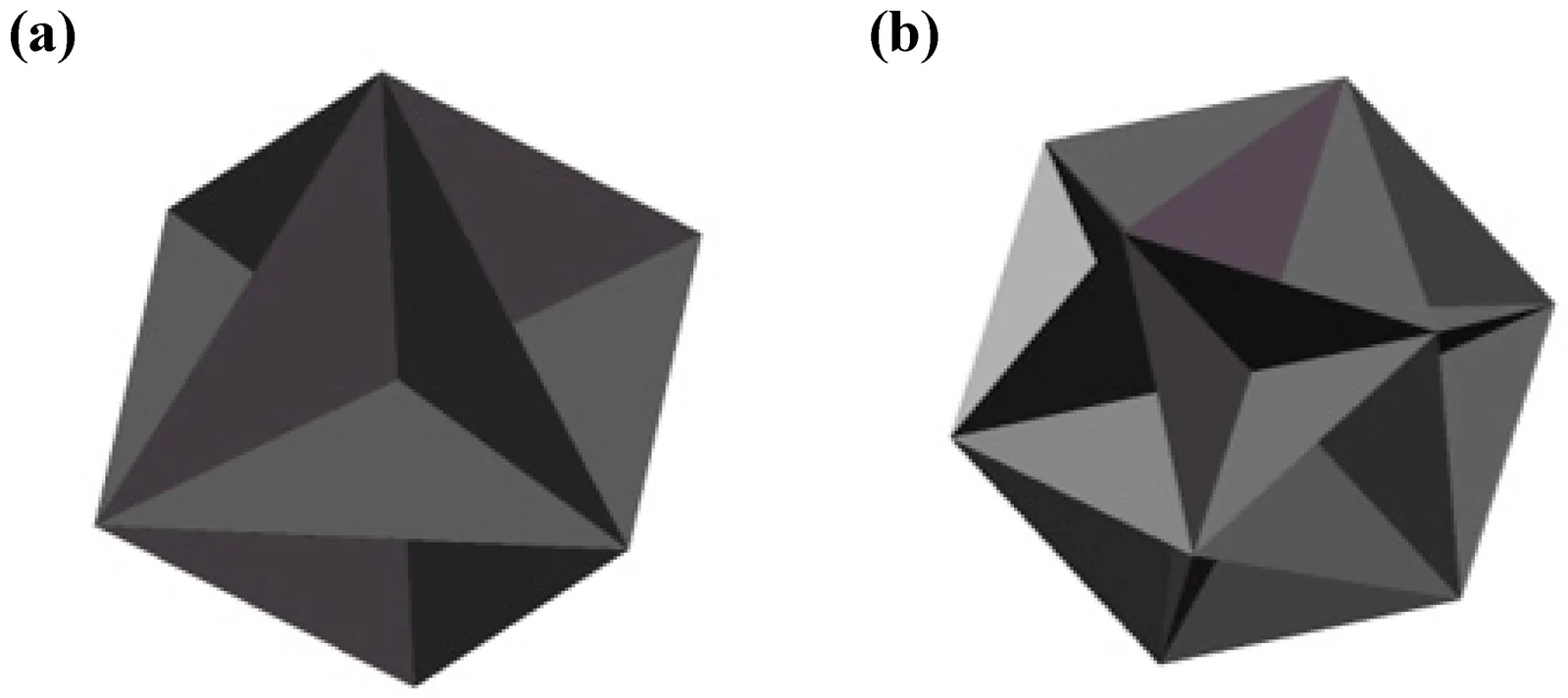
Schematic models of corner reflectors: (**a**) bipyramidal octahedral corner reflector; (**b**) regular icosahedral corner reflector.

As a classical target with a composite multi-triangular-pyramid cavity structure, the corner reflector exhibits considerable geometric complexity. The commonly used corner reflector types mainly include the bipyramidal octahedral corner reflector and the regular icosahedral corner reflector [35,41], whose three-dimensional models are shown in Figure 1. In this study, the regular icosahedral corner reflector, which features greater geometric complexity, is selected as the research object. It is decomposed into twenty regular triangular pyramid structures with open bases, each composed of three congruent isosceles right triangles. Each unit is then equivalently treated as an infrared radiation source cavity with an equilateral triangular aperture and isothermal cavity walls. Starting from a single regular triangular pyramid cavity, the effective emissivity is first derived based on cavity radiation theory. The cavity units are then combined to form the icosahedral corner reflector, and the infrared radiation characteristics of the resulting multi-cavity composite structure are further analyzed and derived.

### 2.1. Cavity Radiation Theory

As a standard radiation source, a blackbody-type radiator serves as the absolute reference for the development of infrared equipment. A blackbody is an idealized concept. According to Kirchhoff’s law, the radiation of a blackbody is equivalent to the radiation inside a closed cavity [42]. In practical applications, blackbody-type radiation sources are generally designed as hollow cavities with an aperture, so that near-blackbody radiation can be achieved [43]. Cavity radiation theory provides the theoretical foundation for the design and application of blackbody radiation sources and enables the effective emissivity of blackbody cavity apertures to be calculated. Two of the most representative approaches are the Gouffé theory, proposed by the French scholar Albert Gouffé in 1945 [44], and the De Vos theory, introduced by J. C. De Vos in 1954 [45]. In deriving his theory, Gouffé adopted several assumptions, including that the cavity walls exhibit diffuse reflection. The resulting method can be used to theoretically calculate the effective aperture emissivity of spherical, cylindrical, and conical cavities [44,46,47,48], and is characterized by a straightforward computational procedure and a clear physical interpretation. By contrast, De Vos did not adopt these assumptions in his derivation, making his theory applicable to cavities and apertures of arbitrary shapes, although its computational procedure is considerably more complex [47,48]. The Gouffé theory is therefore unsuitable for the regular triangular-pyramidal cavity described above. Accordingly, the effective aperture emissivity of this cavity is derived in the present study based on the De Vos theory.

The derivation of the De Vos theory is available in previously published research papers and textbooks. De Vos’s theory first considers the case of a closed cavity and derives the effective emissivity of an arbitrary surface element dω on the cavity wall in an arbitrary direction dσ. For an isothermal cavity, the result is unity, whereas for a non-isothermal cavity, a temperature correction term is obtained as the effective emissivity of the closed cavity. The effective emissivity of an open cavity is then given by subtracting from the effective emissivity of dω in direction dσ under the closed-cavity condition the direct or indirect contribution of dσ to the effective emissivity of dω in that same direction. De Vos’s theory simulates the processes of radiation reflection and escape within the cavity. By calculating the fraction of radiation emitted or reflected through dω that eventually reaches the cavity aperture, the effective emissivity of the cavity can be obtained [47,48,49,50].

The following assumptions are explicitly adopted in the derivation:

(1) Isothermal-cavity assumption: The temperature is assumed to be uniformly distributed over the inner walls of each cavity, and all cavities within the corner reflector are assumed to have the same temperature. This assumption is applicable to the evaluation of the infrared characteristics of corner reflectors under steady-state thermal conditions.
(2) Uniform surface optical properties: The emissivity and reflectivity of the cavity-wall material are assumed to be spatially uniform and independent of position.
(3) Diffuse-gray-surface assumption: The cavity walls are assumed to be ideal diffuse-gray surfaces (Lambertian surfaces). Their emissivity and reflectivity are independent of direction and remain constant with wavelength over the infrared spectral range considered.
(4) Geometrical-optics approximation: The characteristic scale of the cavity-wall surface roughness is assumed to be much larger than the infrared wavelength. Radiative energy transfer can therefore be described using geometrical optics, while wave-optical effects, such as diffraction and interference, are neglected.

### 2.2. Derivation of the Emissivity of an Isothermal Closed Cavity

Consider a surface element dω inside a closed cavity. The radiative power it emits in the direction toward the surface element do is given by:

(1) $$dP_{\omega }^{o}=\varepsilon_{\omega }^{o}L_{bb}(T_{\omega })d\omega \cos\theta_{\omega }^{o}d\Omega_{\omega }^{o}$$

where $\varepsilon_{\omega }^{o}$ is the directional emissivity of the surface element dω toward do, $L_{bb}(T_{\omega })$ is the radiance of a blackbody at temperature $T_{\omega }$, $\theta_{\omega }^{o}$ is the angle between the line connecting dω and do and the outward normal of dω, and $d\Omega_{\omega }^{o}$ is the solid angle subtended by do with respect to dω.

Now consider the case in which the radiation from dω undergoes one reflection inside the cavity. Let another surface element dn be taken on the cavity wall. The radiation emitted from dn, after being reflected by dω, is directed toward the cavity opening do. The radiative power incident from dn onto dω is given by:

(2) $$dP_{n}^{\omega }=\varepsilon_{n}^{\omega }L_{bb}(T_{n})dn\cos\theta_{n}^{\omega }d\Omega_{n}^{\omega }$$

where $\varepsilon_{n}^{\omega }$ is the emissivity of the surface element dn in the direction toward dω, $L_{bb}(T_{n})$ is the blackbody radiance at temperature $T_{n}$, $\theta_{n}^{\omega }$ is the angle between the line connecting dn and dω and the normal to dn, and $d\Omega_{n}^{\omega }$ is the solid angle subtended by dω as seen from dn, which can be expressed as:

(3) $$d\Omega_{n}^{\omega }=\frac{\cos\theta_{\omega }^{n}d\omega }{r_{\omega n}^{2}}$$

In the expression, $r_{\omega n}$ is the distance between dω and dn, and $\theta_{\omega }^{n}$ is the angle between the line connecting dn and dω and the normal to dω. Accordingly, the solid angle subtended by dn with respect to dω can be written as:

(4) $$d\Omega_{\omega }^{n}=\frac{\cos\theta_{n}^{\omega }dn}{r_{\omega n}^{2}}$$

Substituting Equations (3) and (4) into Equation (2), the radiative power incident from dn onto dω can be rewritten as:

(5) $$dP_{n}^{\omega }=\varepsilon_{n}^{\omega }L_{bb}(T_{n})d\omega \cos\theta_{\omega }^{n}d\Omega_{n}^{\omega }$$

Then, the radiative power emitted from dn and reflected by dω into the direction of do can be expressed as:

(6) $$dP_{\omega }^{no}=\varepsilon_{n}^{\omega }L_{bb}(T_{n})d\omega \cos\theta_{\omega }^{n}d\Omega_{\omega }^{n}\rho_{\omega }^{no}d\Omega_{\omega }^{o}$$

where $\rho_{\omega }^{no}$ is the radiative reflectance for radiation from dn reflected by the surface element dω toward do, and $d\Omega_{\omega }^{o}$ denotes the solid angle subtended by do at dω. According to the Helmholtz reciprocity theorem [51]:

(7) $$\cos\theta_{\omega }^{n}\rho_{\omega }^{no}=\cos\theta_{\omega }^{o}\rho_{\omega }^{on}$$

Equation (6) can thus be rewritten as:

(8) $$dP_{\omega }^{no}=\varepsilon_{n}^{\omega }L_{bb}(T_{n})d\omega \cos\theta_{\omega }^{o}d\Omega_{\omega }^{n}\rho_{\omega }^{on}d\Omega_{\omega }^{o}$$

Since the location of the surface element dn on the cavity wall is arbitrary, the contributions of all cavity-wall elements at which a single reflection may occur must be integrated over the hemispherical domain to determine the total contribution in the do direction. For a fixed observation direction do and surface element dω, the term $d\omega \cos\theta_{\omega }^{o}\;d\Omega_{\omega }^{o}$ in the preceding equation is independent of the integration variables and can therefore be taken outside the integral. Accordingly, the reflected radiant power emitted into the solid angle $d\Omega_{\omega }^{o}$ can be expressed as follows:

(9) $$P_{\omega (1)}^{o}=d\omega \cos\theta_{\omega }^{o}d\Omega_{\omega }^{o}\int_{hemisphere}\varepsilon_{n}^{\omega }L_{bb}(T_{n})\rho_{\omega }^{on}d\Omega_{\omega }^{n}$$

On the cavity wall, consider another surface element dm and the case of secondary reflection of infrared radiation on the cavity wall. In this case, the radiation is emitted from dm, incident on dn, reflected by dn toward dω, and then reflected again toward do. First, the radiative power incident from dm to dn is calculated as:

(10) $$dP_{m}^{n}=\varepsilon_{m}^{n}L_{bb}(T_{m})dm\cos\theta_{m}^{n}d\Omega_{m}^{n}$$

where $\varepsilon_{m}^{n}$ is the emissivity of surface element dm in the direction toward dn, $L_{bb}(T_{m})$ is the blackbody radiance at temperature $T_{m}$, $\theta_{m}^{n}$ is the angle between the line connecting dm and dn and the outward normal to dm, and $d\Omega_{m}^{n}$ is the solid angle subtended by dn as seen from dm. Substituting the formula for the solid angle gives:

(11) $$\left\{\begin{array}{c} d\Omega_{m}^{n}=\frac{\cos\theta_{n}^{m}dn}{r_{mn}^{2}}\\ d\Omega_{n}^{m}=\frac{\cos\theta_{m}^{n}dm}{r_{mn}^{2}} \end{array}\right.$$

The radiative power incident from dm to dn can then be rewritten as:

(12) $$dP_{m}^{n}=\varepsilon_{m}^{n}L_{bb}(T_{m})dn\cos\theta_{n}^{m}d\Omega_{n}^{m}$$

Then, the radiative power emitted from dm, incident on dn, and reflected from dn toward dω can be expressed as:

(13) $$dP_{n}^{m\omega }=\varepsilon_{m}^{n}L_{bb}(T_{m})dn\cos\theta_{n}^{m}d\Omega_{n}^{m}\rho_{n}^{m\omega }d\Omega_{n}^{\omega }$$

where $\rho_{n}^{m\omega }$ is the reflectivity for radiation emitted from surface element dm, reflected by dn, and directed toward dω. According to the Helmholtz reciprocity theorem:

(14) $$\cos\theta_{n}^{m}\rho_{n}^{m\omega }=\cos\theta_{n}^{\omega }\rho_{n}^{\omega m}$$

Substituting the solid angle formula yields:

(15) $$\left\{\begin{array}{c} d\Omega_{\omega }^{n}=\frac{\cos\theta_{n}^{\omega }dn}{r_{\omega n}^{2}}\\ d\Omega_{n}^{\omega }=\frac{\cos\theta_{\omega }^{n}d\omega }{r_{\omega n}^{2}} \end{array}\right.$$

Equation (13) can be rewritten as:

(16) $$dP_{n}^{m\omega }=\varepsilon_{m}^{n}L_{bb}(T_{m})d\omega \cos\theta_{\omega }^{n}d\Omega_{n}^{m}\rho_{n}^{\omega m}d\Omega_{\omega }^{n}$$

Then, the radiative power emitted from surface element dm, reflected by dn toward dω, and reflected once more to do, can be written as:

(17) $$dP_{mo}^{n\omega }=\varepsilon_{m}^{n}L_{bb}(T_{m})d\omega \cos\theta_{\omega }^{n}d\Omega_{n}^{m}\rho_{n}^{\omega m}d\Omega_{\omega }^{n}\rho_{\omega }^{no}d\Omega_{\omega }^{o}$$

Similarly, by substituting the Helmholtz reciprocity relation, the above expression can be rewritten as:

(18) $$dP_{mo}^{n\omega }=\varepsilon_{m}^{n}L_{bb}(T_{m})d\omega \cos\theta_{\omega }^{o}d\Omega_{n}^{m}\rho_{n}^{\omega m}d\Omega_{\omega }^{n}\rho_{\omega }^{on}d\Omega_{\omega }^{o}$$

By integrating over dm and dn within the hemispherical space, the radiative power reflected by the cavity from surface element dω toward do after two reflections can be obtained as:

(19) $$P_{\omega (2)}^{o}=d\omega \cos\theta_{\omega }^{o}d\Omega_{\omega }^{o}\iint_{hemisphere}\varepsilon_{m}^{n}L_{bb}(T_{m})d\Omega_{n}^{m}\rho_{n}^{\omega m}d\Omega_{\omega }^{n}\rho_{\omega }^{on}$$

The preceding derivation presents the contributions from self-emission, a single reflection (Equation (9)), and two successive reflections (Equation (19)). Similarly, each additional reflection from the cavity wall introduces one additional level of integration; thus, the contribution involving three reflections contains a triple integral, that involving four reflections contains a quadruple integral, and so forth. Consequently, the total radiant power originating from dω and propagating in the direction do can be expressed as the sum of the self-emission contribution and an infinite series of contributions from successive reflections:

(20) $$P_{\omega }^{o}=d\omega \cos\theta_{\omega }^{o}d\Omega_{\omega }^{o}\left[\varepsilon_{\omega }^{o}L_{bb}\left(T_{\omega }\right)+\int_{hemisphere}\varepsilon_{n}^{\omega }L_{bb}\left(T_{n}\right)\rho_{\omega }^{on}d\Omega_{\omega }^{n}+\iint_{hemisphere}\varepsilon_{m}^{n}L_{bb}\left(T_{m}\right)d\Omega_{n}^{m}\rho_{n}^{\omega m}d\Omega_{\omega }^{n}\rho_{\omega }^{on}+\ldots \right]$$

When the cavity is in a non-isothermal state, the surface elements have different temperatures, and the blackbody radiance $L_{bb}\left(T_{n}\right)$ is therefore no longer constant, making direct integration cumbersome. To separate the temperature-independent terms, a temperature correction factor $C_{n}$ is introduced for normalization:

(21) $$C_{n}=\frac{L_{bb}\left(T_{\omega }\right)-L_{bb}\left(T_{n}\right)}{L_{bb}\left(T_{\omega }\right)}$$

Physically, $C_{n}$ represents the difference between the blackbody radiance of surface element dn and that of the reference surface element dω, normalized by the blackbody radiance at dω. Substituting Equation (21) into Equation (20) allows the temperature-dependent term in each contribution to be separated as follows:

(22) $$P_{\omega }^{o}=L_{bb}\left(T_{\omega }\right)d\omega \cos\theta_{\omega }^{o}d\Omega_{\omega }^{o}\left[\varepsilon_{\omega }^{o}+\int_{hemisphere}\varepsilon_{n}^{\omega }\left(1-C_{n}\right)\rho_{\omega }^{on}d\Omega_{\omega }^{n}+\iint_{hemisphere}\varepsilon_{m}^{n}\left(1-C_{m}\right)d\Omega_{n}^{m}\rho_{n}^{\omega m}d\Omega_{\omega }^{n}\rho_{\omega }^{on}+\ldots \right]$$

By definition, the effective emissivity of surface element dω in the do direction is the ratio of the radiant power actually emitted by the surface element in that direction to the radiant power emitted under the same conditions by a blackbody at the same temperature:

(23) $$\varepsilon_{\omega }^{\prime }=\varepsilon_{\omega }^{o}+\int_{hemisphere}\varepsilon_{n}^{\omega }\left(1-C_{n}\right)\rho_{\omega }^{on}d\Omega_{\omega }^{n}+\iint_{hemisphere}\varepsilon_{m}^{n}\left(1-C_{m}\right)d\Omega_{n}^{m}\rho_{n}^{\omega m}d\Omega_{\omega }^{n}\rho_{\omega }^{on}+\ldots$$

Under the condition that the cavity is isothermal, we have:

(24) $$C_{n}=C_{m}=\ldots =0$$

At this point, the emissivity can be written as:

(25) $$\varepsilon_{\omega }^{\prime }=\varepsilon_{\omega }^{o}+\int_{hemisphere}\varepsilon_{n}^{\omega }\rho_{\omega }^{on}d\Omega_{\omega }^{n}+\iint_{hemisphere}\varepsilon_{m}^{n}d\Omega_{n}^{m}\rho_{n}^{\omega m}d\Omega_{\omega }^{n}\rho_{\omega }^{on}+\ldots$$

This expression indicates that if radiation is incident from do onto dω, the portion absorbed at dω is $\varepsilon_{\omega }^{o}$; the portion absorbed after one reflection is represented by the second term in Equation (25); the portion absorbed after two reflections is represented by the third term in Equation (25); and so on. In this way, all subsequent radiation reflected by the cavity walls is eventually absorbed. Therefore, the emissivity of an isothermal closed cavity is equal to 1.

### 2.3. Derivation of the Effective Emissivity of an Isothermal Open Cavity

Opening an aperture at do in an isothermal closed cavity has an effect on the effective emissivity of the surface element dω equivalent to subtracting, under the closed-cavity condition, the direct and indirect influences of do on dω. Similarly, the total power emitted from dω toward do can be expressed as follows: the radiation power incident from dω onto do under the closed condition, minus the radiation power emitted by do and reflected by dω back to do, and minus the radiation power emitted by do, reflected by the entire cavity onto dω, and then reflected back to do. The detailed derivation is as follows:

Since the emissivity of an isothermal closed cavity is 1, the radiative power incident from dω onto do is:

(26) $$dP_{\omega }^{o}=L_{bb}(T)d\omega \cos\theta_{\omega }^{o}d\Omega_{\omega }^{o}$$

where T is the temperature of the inner wall of the cavity. Under the closed-cavity condition, the radiative power emitted from do, reflected by the surface element dω, and then incident again on do is:

(27) $$dP_{\omega }^{oo}=L_{bb}(T)do\cos\theta_{o}^{\omega }d\Omega_{o}^{\omega }\rho_{\omega }^{oo}d\Omega_{\omega }^{o}$$

As in the derivation in Section 2.2, by substituting the solid-angle relation, the above expression can be rewritten as:

(28) $$dP_{\omega }^{oo}=L_{bb}(T)d\omega \cos\theta_{\omega }^{o}d\Omega_{\omega }^{o}\rho_{\omega }^{oo}d\Omega_{\omega }^{o}$$

Take another surface element dp. Consider the radiation emitted from do, incident on dp, reflected to dω, and then incident on do again. Similarly, the corresponding radiative power can be expressed as:

(29) $$dP_{oo}^{p\omega }=L_{bb}(T)do\cos\theta_{o}^{p}d\Omega_{o}^{p}\rho_{p}^{o\omega }d\Omega_{p}^{\omega }\rho_{\omega }^{po}d\Omega_{\omega }^{o}$$

By substituting the solid-angle formula:

(30) $$\left\{\begin{array}{c} d\Omega_{o}^{p}=\frac{\cos\theta_{p}^{o}dp}{r_{op}^{2}}\\ d\Omega_{p}^{o}=\frac{\cos\theta_{o}^{p}do}{r_{op}^{2}} \end{array}\right.$$

and the Helmholtz reciprocity relation:

(31) $$\cos\theta_{p}^{o}\rho_{p}^{o\omega }=\cos\theta_{p}^{\omega }\rho_{p}^{\omega o}$$

Equation (29) can thus be rewritten as:

(32) $$dP_{oo}^{p\omega }=L_{bb}(T)dp\cos\theta_{p}^{\omega }d\Omega_{p}^{o}\rho_{p}^{\omega o}d\Omega_{p}^{\omega }\rho_{\omega }^{po}d\Omega_{\omega }^{o}$$

Similarly, by substituting the solid-angle formula again and applying the Helmholtz reciprocity theorem, the final expression for the radiative power is obtained as:

(33) $$dP_{oo}^{p\omega }=L_{bb}(T)d\omega \cos\theta_{\omega }^{o}d\Omega_{\omega }^{o}\rho_{p}^{\omega o}d\Omega_{p}^{o}\rho_{\omega }^{op}d\Omega_{\omega }^{p}$$

By integrating over the portion of the hemisphere subtended by the differential area element dp, excluding the cavity aperture, one obtains—under closed conditions—the radiative power emitted from do, reflected by the cavity wall to dω, and then reflected back to do:

(34) $$P_{\omega (1)}^{oo}=L_{bb}(T)d\omega \cos\theta_{\omega }^{o}d\Omega_{\omega }^{o}\int_{hemisphere-do}\rho_{p}^{\omega o}d\Omega_{p}^{o}\rho_{\omega }^{op}d\Omega_{\omega }^{p}$$

Based on the preceding analysis, in an open cavity, the radiant power actually transferred from surface element dω toward the aperture do is equal to the radiant power under the corresponding closed-cavity condition (Equation (26)), minus the following two contributions lost because of the presence of the aperture: (1) the radiation emitted by do and reflected directly back to do by dω (Equation (28)); (2) the radiation emitted by do, reflected by the other cavity walls, and ultimately returned to do (Equation (34)). Therefore, the effective radiant power transferred from surface element dω to the cavity aperture is given by the difference among these three terms:

(35) $$dP_{\omega }^{o}=L_{bb}(T)d\omega \cos\theta_{\omega }^{o}d\Omega_{\omega }^{o}\left[1-\rho_{\omega }^{oo}d\Omega_{\omega }^{o}-\int_{hemisphere-do}\rho_{p}^{\omega o}d\Omega_{p}^{o}\rho_{\omega }^{op}d\Omega_{\omega }^{p}\right]$$

According to the definition of emissivity, in an arbitrary cavity with an opening, the effective emissivity of the surface element dω in the direction toward the aperture can be written as:

(36) $$\varepsilon_{\omega }=1-\rho_{\omega }^{oo}d\Omega_{\omega }^{o}-\int_{hemisphere-do}\rho_{p}^{\omega o}d\Omega_{p}^{o}\rho_{\omega }^{op}d\Omega_{\omega }^{p}$$

### 2.4. Derivation of the Effective Emissivity of the Aperture of an Isothermal Regular Triangular Pyramid Cavity

Section 2.2 and Section 2.3 briefly reviewed the fundamental derivations of effective emissivity for closed and open cavities within the framework of classical cavity radiation theory [49,50]. To the best of our knowledge, no analytical approximation for the effective emissivity of a regular triangular-pyramidal cavity has yet been presented in existing textbooks. Therefore, building on the preceding theoretical framework, this section derives an equivalent model for such a cavity for the first time.

**Figure 2:**
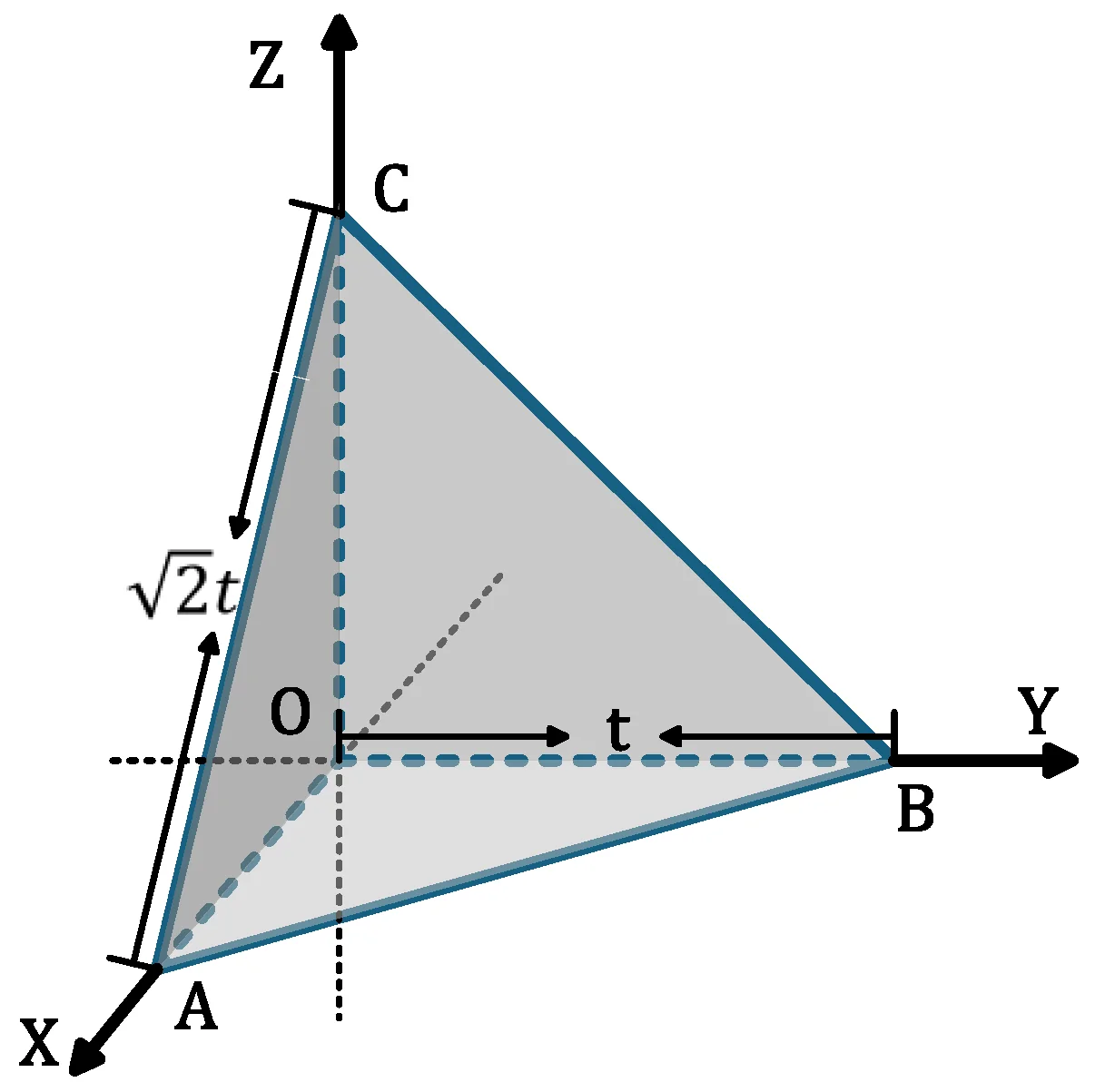
Schematic diagram of the Cartesian coordinate system for a regular triangular pyramid.

A three-dimensional Cartesian coordinate system is established for the regular triangular pyramid cavity, with the pyramid apex coinciding with the origin O. The three lateral edges OA, OB, and OC are aligned with the x-, y-, and z-axes, respectively. Let the lateral edge length be t. The base of the pyramid is an equilateral triangular aperture with side length $\sqrt{2}\;t$, as shown in Figure 2.

**Figure 3:**
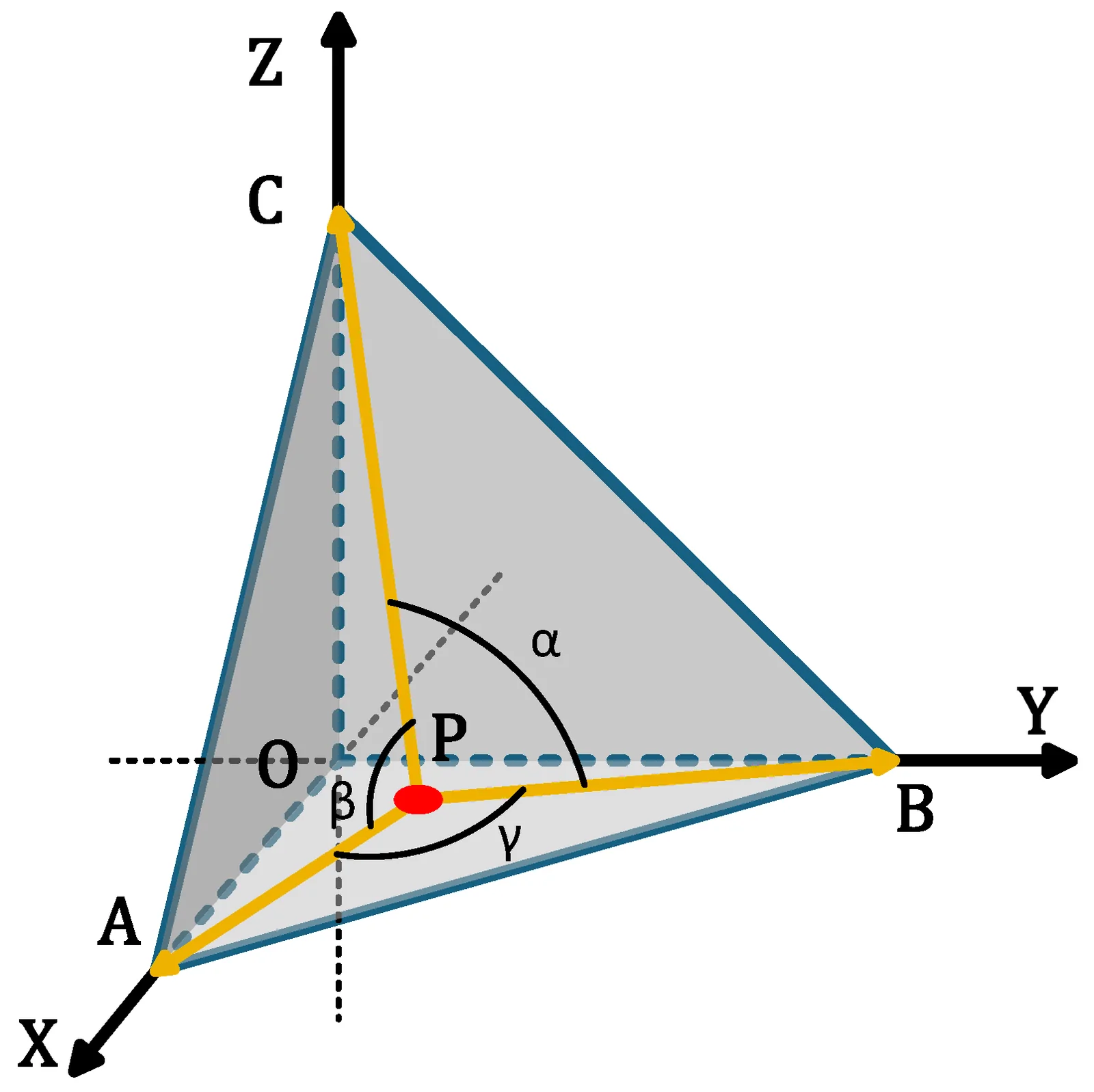
Schematic diagram of the first-order approximation method for effective emissivity calculation.

Consider an arbitrary surface element dp on the inner wall of the cavity. Taking the case where the element lies in the AOB plane as an example, let the coordinates of its center point be P(x,y,0), as shown in Figure 3. According to Equation (36), the derivation begins with the solid angle subtended by the cavity aperture at dp. In the present model, the aperture is an equilateral triangle of specified dimensions. At an arbitrary observation point in space, the solid angle subtended by this planar triangle is equivalent to the area of a spherical triangle on a unit sphere centered at the observation point. Accordingly, L’Huilier’s theorem, a classical result in spherical trigonometry, is introduced to calculate this solid angle. Proposed by the Swiss mathematician Simon L’Huilier, the theorem provides a standard method for calculating the area of a spherical triangle and is described extensively in numerous geometry textbooks [52,53]. It has been widely applied in fields including spherical geometry [54], radiative transfer [55], and astrophysics [56]. By using the angles between the lines of sight from the observation point to the three vertices of the planar triangle, L’Huilier’s theorem enables the solid angle to be calculated directly, thereby avoiding complicated integration and significantly improving computational efficiency.

The vectors from point P to the three vertices A, B, and C of the cavity aperture are respectively expressed as:

(37) $$\left\{\begin{array}{l} \vec{PA}=(t-x,-y,0)\\ \vec{PB}=(-x,t-y,0)\\ \vec{PC}=\left(-x,-y,t\right) \end{array}\right.$$

Taking the observation point P as the center of a unit sphere, the equilateral triangular aperture is mapped onto an equivalent spherical triangle. Let α, β, and γ denote the angles between $\vec{PB}$ and $\vec{PC}$, between $\vec{PA}$ and $\vec{PC}$, and between $\vec{PA}$ and $\vec{PB}$, respectively. Once these angles have been determined directly through vector operations, the semiperimeter of the spherical triangle can be calculated as follows:

(38) $$s=\frac{\alpha +\beta +\gamma }{2}$$

According to L’Huilier’s formula, the spherical excess can be calculated, which is numerically equal to the solid angle subtended by the cavity aperture at point P. It is expressed as follows:

(39) $$\Omega_{p}=4\tan^{-1}\sqrt{\tan\frac{s}{2}\tan\frac{s-\alpha }{2}\tan\frac{s-\beta }{2}\tan\frac{s-\gamma }{2}}$$

For the infinitesimal surface element dp, the variation in viewing angle within the element can be approximately neglected. Therefore, in this study, the solid angle at the center point P of the surface element is used as an equivalent representation of the solid angle over the entire element. Thus, the solid angle subtended by the triangular aperture at the surface element dp can be written as:

(40) $$d\Omega_{p}^{o}=\Omega_{p}dp$$

Due to the differences in relative position, viewing distance, and spatial angle between each surface element on the cavity wall and the aperture, the solid angle subtended by the aperture varies significantly across the inner wall. Therefore, the local emissivity obtained from the solid angle at a single position cannot represent the overall cavity behavior. To obtain the equivalent average solid angle of the aperture with respect to the entire cavity wall, the local solid angles over the whole domain should first be integrated and summed to determine the total solid angle. This total is then divided by the total area, converting it into the average solid angle subtended by the aperture per unit area of the cavity wall. This average value can then be used as a constant geometric parameter in the calculation of the effective emissivity of the aperture.

For a regular triangular pyramid, the three lateral faces are congruent and exhibit strict symmetry. Therefore, integrating the solid angle subtended by the aperture at dp over cavity wall AOB yields the same result as performing the solid-angle integration over surface elements on faces AOC and BOC, respectively. Hence, the average solid angle subtended by the aperture per unit area of the cavity wall can be expressed as:

(41) $$d\Omega =\frac{1}{\frac{3}{2}t^{2}}\times 3\times 4\int_{0}^{t}dx\int_{0}^{t-x}\tan^{-1}\sqrt{\tan\frac{s}{2}\tan\frac{s-\alpha }{2}\tan\frac{s-\beta }{2}\tan\frac{s-\gamma }{2}}dy$$

Based on Equation (36), the first-order approximation for the effective emissivity of the aperture of a regular triangular-pyramidal cavity with an equilateral triangular opening can be obtained as:

(42) $$\varepsilon_{1}=1-\varepsilon_{1}^{\prime }=1-\frac{d\Omega }{2\pi }\rho$$

Here, ρ denotes the average reflectivity of the cavity wall, and $\varepsilon_{1}^{\prime }$ is the first-order approximation term of the effective emissivity. This expression completes the calculation of the fraction of radiation that, under the closed-cavity condition, returns to the aperture after one reflection from the cavity wall, or equivalently, under the open-cavity condition, the fraction of wall radiation that escapes from the cavity. On this basis, the effective emissivity of the cavity aperture can be approximately obtained.

**Figure 4:**
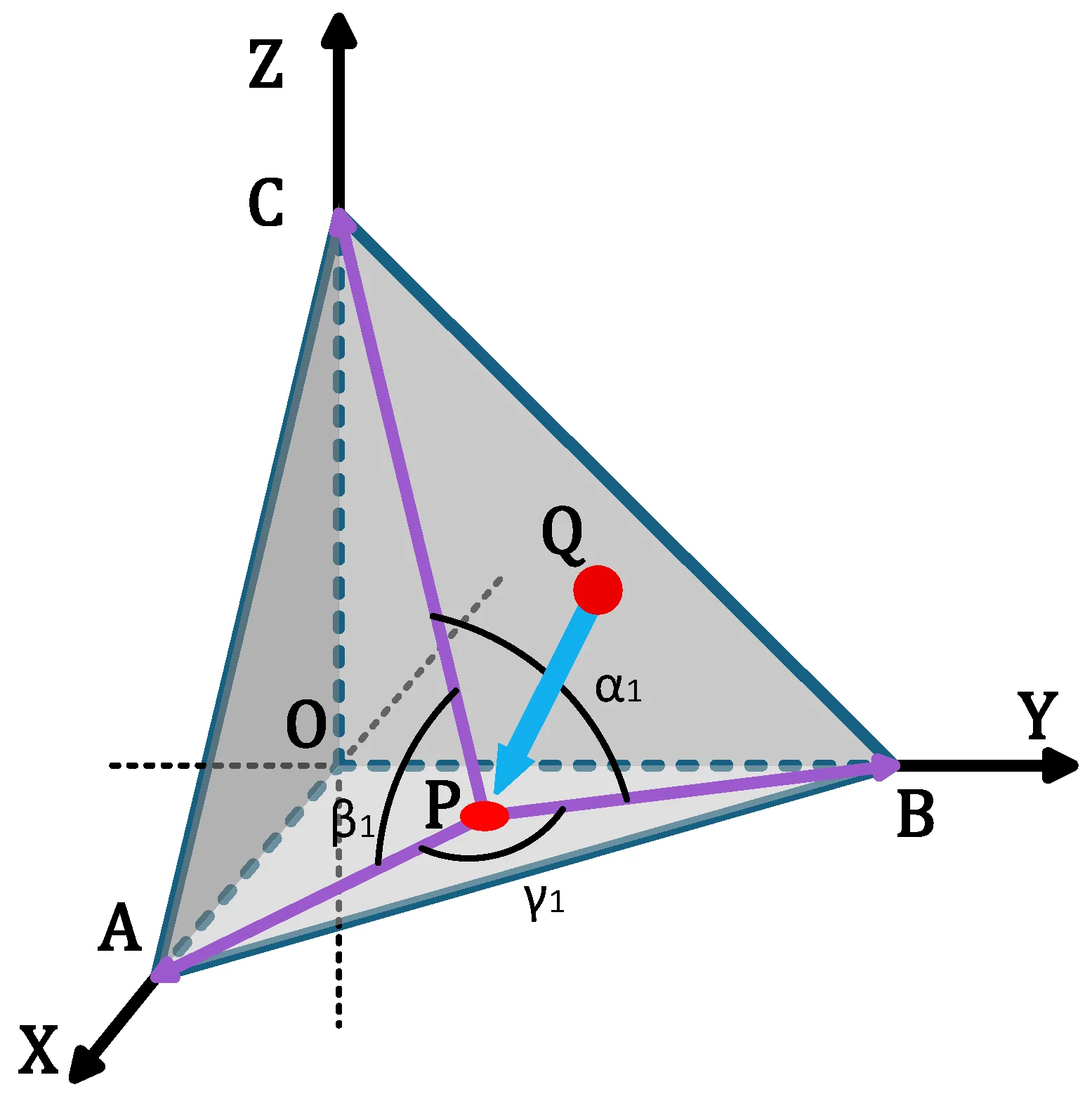
Schematic diagram of the second-order approximation method for effective emissivity calculation.

According to De Vos theory, the effective emissivity can be expanded as a sum of contributions corresponding to successive orders of reflection. The first-order approximation accounts only for the fraction of radiation that escapes directly through the cavity aperture. To obtain a more accurate estimate of the effective emissivity, the contribution of radiation that escapes through the aperture after undergoing one reflection from the cavity wall must also be evaluated; this contribution constitutes the second-order approximation. Consider an arbitrary surface element dq on the cavity wall. Taking dq on face BOC as an example, let its center be $Q\left(0,y_{2},z_{2}\right)$. Surface element dp lies on face AOB, with its center denoted by $P\left(x_{1},y_{1},0\right)$, as shown in Figure 4.

For a regular triangular-pyramidal cavity, the probability that radiation emitted by surface element dp escapes through the aperture after being reflected by surface element dq depends on three geometric factors. The first is the weighting associated with emission from dp toward the aperture, which is proportional to the solid angle subtended by the aperture at dp. The second is the probability that dq receives radiation from dp, which is proportional to the solid angle subtended by dp at dq. The third is the weighting associated with reflection from dq toward the aperture, which is proportional to the solid angle subtended by the aperture at dq.

Let the vector from point Q to point P be denoted by $\vec{r}$. Then, the angle between line segment PQ and the normal to the plane containing point P can be expressed as:

(43) $$\theta =\cos^{-1}\frac{\vec{n}\cdot \vec{r}}{\left|n\right|\left|r\right|}=\frac{z_{2}}{\sqrt{x_{1}^{2}+{\left(y_{1}-y_{2}\right)}^{2}+z_{2}^{2}}}$$

Here, $\vec{n}$ is the normal vector of plane AOB. Similarly, for points within surface element dq, the variation in the viewing angle of surface element dp can be neglected. Therefore, the solid angle subtended by dp at different points within dq can be approximated as equal to the solid angle subtended by dp at point Q. According to the basic definition of solid angle, the solid angle subtended by dp with respect to dq can be written as:

(44) $$d\Omega_{q}^{p}=\frac{dp\cos\theta }{r_{pq}^{2}}dq$$

Here, $r_{pq}$ is the distance between points P and Q. When calculating the solid angle subtended by the cavity aperture with respect to surface element dp, the conditions still satisfy the applicability requirements of L’Huilier’s formula. Therefore, L’Huilier’s formula can again be used to calculate the solid angle subtended by the aperture at point P. First, the vectors from point P to the three vertices A, B, and C of the aperture are calculated as follows:

(45) $$\left\{\begin{array}{l} \vec{PA}=(t-x_{1},-y_{1},0)\\ \vec{PB}=(-x_{1},t-y_{1},0)\\ \vec{PC}=\left(-x_{1},-y_{1},t\right) \end{array}\right.$$

Taking observation point P as the center of a unit sphere, aperture ABC is equivalently mapped onto a spherical triangle. Let $\alpha_{1}$ denote the angle between $\vec{PB}$ and $\vec{PC}$, $\beta_{1}$ the angle between $\vec{PA}$ and $\vec{PC}$, and $\gamma_{1}$ the angle between $\vec{PA}$ and $\vec{PB}$. These angles are the three central angles corresponding to the sides of the spherical triangle, whose semiperimeter is given by:

(46) $$s_{1}=\frac{\alpha_{1}+\beta_{1}+\gamma_{1}}{2}$$

According to L’Huilier’s formula, the solid angle subtended by aperture ABC with respect to surface element dp is:

(47) $$d\Omega_{p}^{ABC}=4\tan^{-1}\sqrt{\tan\frac{s_{1}}{2}\tan\frac{s_{1}-\alpha_{1}}{2}\tan\frac{s_{1}-\beta_{1}}{2}\tan\frac{s_{1}-\gamma_{1}}{2}}dp$$

By integrating over dq on plane BOC, the total solid angle subtended by wall AOB with respect to wall BOC, denoted as $\Omega_{BOC}^{AOB}$, can be obtained. Owing to the symmetry between planes AOB and AOC, the total solid angles subtended by these two walls with respect to wall BOC, namely $\Omega_{BOC}^{AOB}$ and $\Omega_{BOC}^{AOC}$, are equal in value. Consequently, the average solid angle per unit area subtended by these two walls with respect to wall BOC is also equal.

Similarly, based on the symmetry among the three walls of the regular triangular pyramid cavity, it can be concluded that the average solid angle per unit area subtended by any one wall to another wall inside the cavity is the same for all wall pairs. Likewise, the solid angles subtended by the aperture with respect to the three walls are also equal in magnitude.

Therefore, for a regular triangular pyramid cavity with an open base, the second-order approximation of the effective emissivity of the cavity aperture per unit area of the cavity wall can be expressed as:

(48) $$\varepsilon_{2}^{\prime }=\frac{3}{\frac{3}{2}t^{2}}\iint_{BOC}\iint_{AOB}\rho^{2}\frac{2d\Omega_{q}^{p}}{2\pi }\frac{d\Omega_{p}^{ABC}}{2\pi }=\frac{3\rho^{2}}{\frac{3}{2}t^{2}}\int_{0}^{z_{2}}\int_{0}^{t-x_{2}}\int_{0}^{y_{1}}\int_{0}^{t-x_{1}}\frac{4z_{2}\tan^{-1}\sqrt{\tan\frac{s_{1}}{2}\tan\frac{s_{1}-\alpha_{1}}{2}\tan\frac{s_{1}-\beta_{1}}{2}\tan\frac{s_{1}-\gamma_{1}}{2}}dx_{1}dy_{1}dz_{2}dy_{2}}{4\pi^{2}{\left[x_{1}^{2}+{\left(y_{1}-y_{2}\right)}^{2}+z_{2}^{2}\right]}^{\frac{3}{2}}}$$

The second-order approximation can be written as:

(49) $$\varepsilon_{2}=1-\varepsilon_{1}^{\prime }-\varepsilon_{2}^{\prime }$$

The derivation procedure in this section, based on cavity radiation theory and theorems of spherical trigonometry, for obtaining an approximate expression of the effective emissivity of the aperture of a regular triangular pyramidal cavity can be extended to a general triangular pyramidal cavity. Since a general triangular pyramidal cavity does not possess the rotational symmetry of a regular triangular pyramidal cavity, the solid-angle calculation should be carried out using L’Huilier’s formula by integrating, over the plane of the wall on which each surface element lies, the solid angle subtended at each wall element by the aperture and by the other two cavity walls, respectively.

### 2.5. Equivalent Modeling of the Infrared Radiation Characteristics of the Corner Reflector

The average effective emissivity of the regular triangular pyramidal cavity derived in the preceding section has, through full-area integration and averaging, already eliminated the radiative nonuniformity among the differential wall elements. It should be noted, however, that when a single regular triangular pyramidal cavity is observed from different viewing angles, the cavity-wall region within the field of view and the corresponding solid-angle distribution of the aperture vary due to the cavity geometry, resulting in spatial differences in the local effective emissivity. Therefore, if the full-area average emissivity is directly applied to a single isolated regular triangular pyramidal cavity, a certain radiative calculation error may be introduced because of the viewing-angle deviation.

However, in the structure of a regular icosahedral corner reflector, the twenty cavities are uniformly distributed in all spatial directions. From a statistical point of view, the imaging field of view corresponding to any far-field observation angle simultaneously covers multiple cavity units with relatively stronger radiation, weaker radiation, and intermediate radiative characteristics. As a result, the random viewing-angle error introduced by any individual cavity tends to cancel out. Under this condition, substituting the average effective emissivity does not introduce a significant systematic bias and thus ensures the accuracy of the model.

There is no aperture-to-aperture line of sight among the regular triangular pyramid cavities in the icosahedral corner reflector; therefore, no cross-radiative coupling occurs, and the radiation from each cavity can be regarded as mutually independent. Moreover, since all cavities are identical in structure and material properties, under the condition of a uniform and constant overall temperature, the radiative emission behavior of each aperture and the proportion of energy escaping through the aperture are highly consistent, with no local discrepancy in radiative characteristics. For a regular icosahedral corner reflector, the projected area at any observation angle is exactly equal to that of a standard solid regular icosahedron of the same volume. By assigning to every surface element of the corner reflector the effective emissivity of a unit-area wall element of the regular triangular pyramid cavity with respect to its aperture, the far-field infrared radiation characteristics of the reflector, when observed from a distance several tens of times greater than its width, are determined solely by the target’s projected contour, surface emissive capability, and thermodynamic temperature. The complex cavity structure on the reflector does not alter the geometric contribution to far-field radiation.

**Figure 5:**
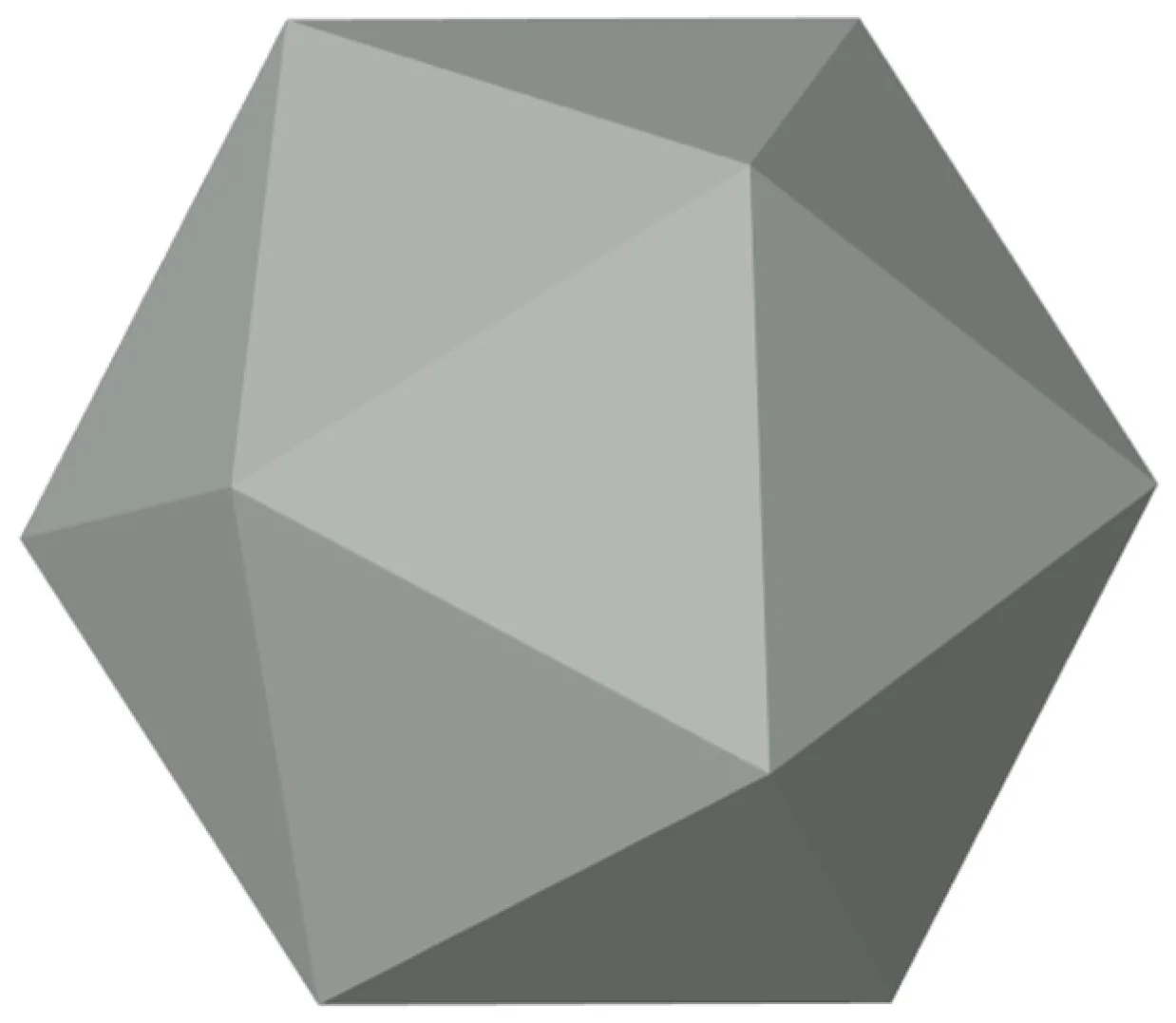
Equivalent regular icosahedron model.

To simplify the infrared radiation calculation of this complex multi-cavity structure while maintaining computational accuracy, this study adopts an equivalent surface-source modeling method to represent the radiative characteristics of the icosahedral corner reflector target. Specifically, the composite structure formed by 20 identical triangular pyramid cavities is replaced by an equivalent solid standard regular icosahedron with the same external contour and uniform surface radiative properties, as shown in Figure 5. The effective emissivity obtained for a single triangular pyramid cavity through full-area integral averaging is uniformly assigned to all outer surfaces of the equivalent regular icosahedron, thereby enabling a simplified calculation of the infrared radiation of the complex multi-cavity structure.

Planck’s law states that the radiative capability of a blackbody is determined by its temperature and wavelength. According to Planck’s formula, the spectral radiant exitance of a blackbody, $M_{\lambda bb}$ is given by:

(50) $$M_{\lambda bb}=\frac{2\pi hc^{2}}{\lambda^{5}}\cdot \frac{1}{e^{\frac{hc}{\lambda K_{B}T}}-1}=\frac{c_{1}}{\lambda^{5}}\cdot \frac{1}{e^{c_{2}/\lambda T}-1}$$

where λ is the wavelength, T is the thermodynamic temperature, c is the speed of light, h is Planck’s constant, $K_{B}$ is Boltzmann’s constant, $c_{1}$ is the first radiation constant, and $c_{2}$ is the second radiation constant. Based on the equivalent surface-source treatment method, the radiative characteristics of the regular icosahedral corner reflector can be equivalently represented by those of a regular icosahedron. Accordingly, its spectral radiant exitance M(λ,T) at a given wavelength λ and temperature T can be expressed as:

(51) $${M\left(\lambda ,T\right)=\varepsilon }_{2}\cdot \frac{c_{1}}{\lambda^{5}}\cdot \frac{1}{e^{c_{2}/\lambda T}-1}$$

The total radiant exitance over a given wavelength band can be obtained by integrating Equation (52) with respect to wavelength λ:

(52) $$M\left(\lambda ,T\right)=\int_{\lambda_{1}}^{\lambda_{2}}\varepsilon_{2}\cdot \frac{c_{1}}{\lambda^{5}}\cdot \frac{1}{e^{c_{2}/\lambda T}-1}d\lambda$$

The surface material of the corner reflector is a flexible metallic fabric woven from metal fibers and characterized by microscopically rough textures. Its infrared radiation is dominated by diffuse reflection, and the radiative energy distribution in the hemispherical space tends to be uniform, without significant directional specular radiation effects. Therefore, it satisfies the isotropic diffuse radiation characteristics of a Lambertian emitter. Accordingly, the radiance of the regular icosahedral target can be calculated based on Lambert’s cosine law, from which its spectral radiant intensity is obtained as:

(53) $$I_{\lambda }=L_{\lambda }A=\frac{M(\lambda ,T)A}{\pi }$$

where $I_{\lambda }$ is the spectral radiant intensity of the target, with unit W/(sr⋅μm); $L_{\lambda }$ is the spectral radiance, with unit $W/(sr\cdot \mu m\cdot m^{2})$; and A is the projected area of the target at the detector viewing angle. In addition, under ideal conditions where atmospheric absorption and scattering attenuation are neglected, and for an observation scenario in which the detector-to-target distance is several tens of times larger than the target size, the target can be regarded as a point source. Then, according to the target radiance, combined with the target projected area facing the detector and the detection range, the irradiance at the detector entrance pupil can be calculated using the radiative transfer relation as:

(54) $$E=\frac{I_{\lambda }}{R^{2}}=\frac{M\left(\lambda ,T\right)A}{\pi R^{2}}$$

where E is the irradiance of the target at the detector, with unit $W/(\mu m\cdot m^{2})$, and R is the distance between the detection point and the target.

## 3. Simulation Verification and Results Analysis

### 3.1. Basic Principles of RMCM and IRTM

The reverse Monte Carlo method (RMCM) and the ray tracing method (RTM) are currently recognized as mainstream approaches in the field of infrared signature simulation. RMCM, based on unbiased statistical sampling, is well suited for handling multiple scattering and participating media [57,58]; RTM, based on geometrical optics, can efficiently simulate direct radiation and specular reflection [59]. In this section, infrared radiation characteristic simulations of corner reflectors are carried out based on RMCM and the improved ray tracing method (IRTM), in order to verify the previously derived effective emissivity calculation method for a regular triangular pyramid cavity and the equivalent calculation model for the radiation characteristics of an icosahedral corner reflector.

The Monte Carlo method (MCM) treats a series of physical subprocesses in infrared radiative transfer—such as emission, absorption, reflection, and scattering—as random interactions between a large number of independent rays and the medium or wall surfaces, and statistically solves the radiative transfer equation [60,61]. RMCM further applies the reciprocity principle of radiative transfer by setting the tracing starting point at the detector rather than at the radiation source. Specifically, N characteristic rays are emitted in the reverse direction from the detector within the field-of-view solid angle. By tracing each ray backward along its path, its final destination is determined, i.e., whether it is absorbed by the medium or by a solid wall. The blackbody radiance corresponding to the local temperature at the absorption point is then taken as that ray’s radiative contribution to the detector. Finally, averaging over all rays yields the spectral irradiance at the detector and the target radiation intensity. The infrared radiation intensity based on RMCM is expressed as follows:

(55) $$I=A\sum_{i=1}^{N}\frac{L_{b\lambda }(i)}{N}$$

where N is the total number of rays, A is the equivalent area contribution given by the projection of the total area of object-plane pixels in the detector direction, and $L_{b\lambda }(i)$ is the blackbody spectral radiance calculated from the temperature at the absorption point of the i-th ray. Since the probability of ray absorption has already been governed by the medium absorptivity or wall emissivity [62], the contribution of the absorption point can be taken directly as the blackbody radiance at its local temperature. It can thus be seen that RMCM simulation essentially transforms the problem of calculating the target radiation intensity at the detector into the problem of determining the absorption points of randomly traced rays. Therefore, there is no need to explicitly compute the effective radiation term, which greatly simplifies the problem.

The core task for each ray in RMCM is trajectory tracing within a CFD unstructured mesh. A ray starts from the detector and first searches for the incident surface element on the transparent boundary of the computational domain to determine the entry cell. After entering the domain, RTM is used to advance the ray cell by cell: among all boundary surface elements of the current cell, the surface element intersected by the outgoing ray is identified, and then the ray transitions to the adjacent cell using mesh topology relations. This process is repeated until the ray is absorbed by the medium, absorbed by a solid wall, or escapes from the computational domain [63]. The ray–surface intersection test is performed as follows: let P be the intersection point between the ray and the plane containing the surface element; let the vertices of the surface element be denoted in counterclockwise order by i; define ${\vec{r}}_{Pi}\;(i=1,\ldots ,n)$ as the vectors originating from P and terminating at each vertex of the surface element. The criterion is given by:

(56) $$\beta_{i}=\left({\vec{r}}_{P(i\%n)}\times {\vec{r}}_{P(\left(i+1\right)\%n)}\right)\cdot \vec{n}\;(i=1\ldots n)$$

where $\vec{n}$ is the normal vector of the surface element. If all $\beta_{i}$ in the above expression are nonnegative for every vertex of the surface element, then the ray is confirmed to intersect that surface element. However, because of the large disparity between the detection distance and the CFD mesh size, the calculation of the ray–surface intersection coordinates is significantly affected by floating-point truncation errors. This deviation may lead to two types of failure. First, the computed intersection point may fall outside the surface-element boundary, so that not all $\beta_{i}$ are nonnegative, making it impossible to identify the outgoing surface element. Second, the ray may simultaneously satisfy the intersection conditions for two adjacent surface elements, resulting in an incorrect choice of the outgoing surface element. Since there is a probability of failure each time a ray traverses a control volume, and this probability accumulates with the number of traversed control volumes, some rays eventually become “failed rays” and cannot be traced successfully to completion. Because the mesh near high-temperature components is usually finer and less orthogonal, failed rays tend to concentrate in these regions, causing the high-temperature contribution to be underrepresented in the statistical results and thereby introducing systematic bias.

For the improved ray tracing method (IRTM), the decision model is given by:

(57) $$\kappa_{i}=\left\{\begin{array}{c} \left({\vec{r}}_{P_{0}\left(i\%n\right)}\times {\vec{r}}_{P_{0}\left(\left(i+1\right)\%n\right)}\right)\cdot \vec{r}\;\;\vec{r}\cdot \vec{n}<0\\ \left({\vec{r}}_{P_{0}\left(\left(i+1\right)\%n\right)}\times {\vec{r}}_{P_{0}\left(i\%n\right)}\right)\cdot \vec{r}\;\;\vec{r}\cdot \vec{n}>0 \end{array}\right.\;(i=1,\ldots ,n)$$

In IRTM, the method no longer relies on the exact coordinates of the intersection point. Instead, taking the known incident point $P_{0}$ in the current cell as the reference, it determines whether the ray direction vector is enclosed by the vectors connecting $P_{0}$ to the vertices of a surface element. In Equation (57), ${\vec{r}}_{P_{0}i}$ denotes the vector from $P_{0}$ to each vertex of the surface element, and $\vec{r}$ is the ray direction vector. If $\kappa_{i}$ in the above expression is nonnegative for all vertices, then the ray is considered to intersect that surface element; otherwise, the intersection point does not lie within the surface element. Since the ray direction vector is not affected by truncation errors, and all computations are performed at the scale of the control-volume characteristic dimensions, the accuracy of the intersection test is greatly improved.

RMCM has been successfully applied in infrared signature simulations of various types of targets, and IRTM improves the original simulation framework further, enhancing computational accuracy [64]. Therefore, using the RMCM-IRTM simulation method for comparative validation has a rigorous physical basis and sufficient accuracy assurance, and can reliably be employed to evaluate the effectiveness of the method proposed in this paper.

### 3.2. Simulation Framework and Parameter Settings

**Figure 6:**
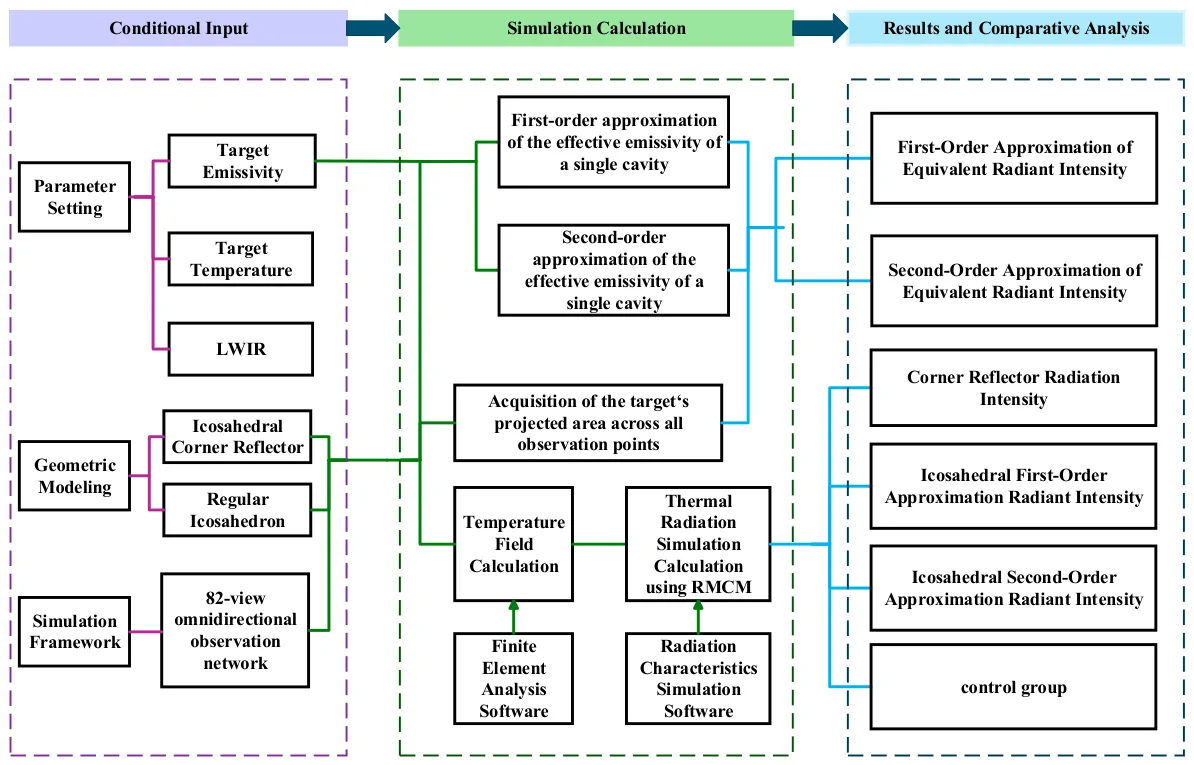
Schematic diagram of the experimental procedure.

In this section, infrared characteristic calculation experiments are carried out using a regular icosahedral corner reflector and a regular icosahedron as the study objects. Under specified initial temperatures and wavelength bands, their infrared radiation characteristics are calculated respectively based on the effective-emissivity approximate formula derived above and the RMCM simulation software, and the resulting data are compared and analyzed. The overall experimental framework is shown in Figure 6.

**Figure 7:**
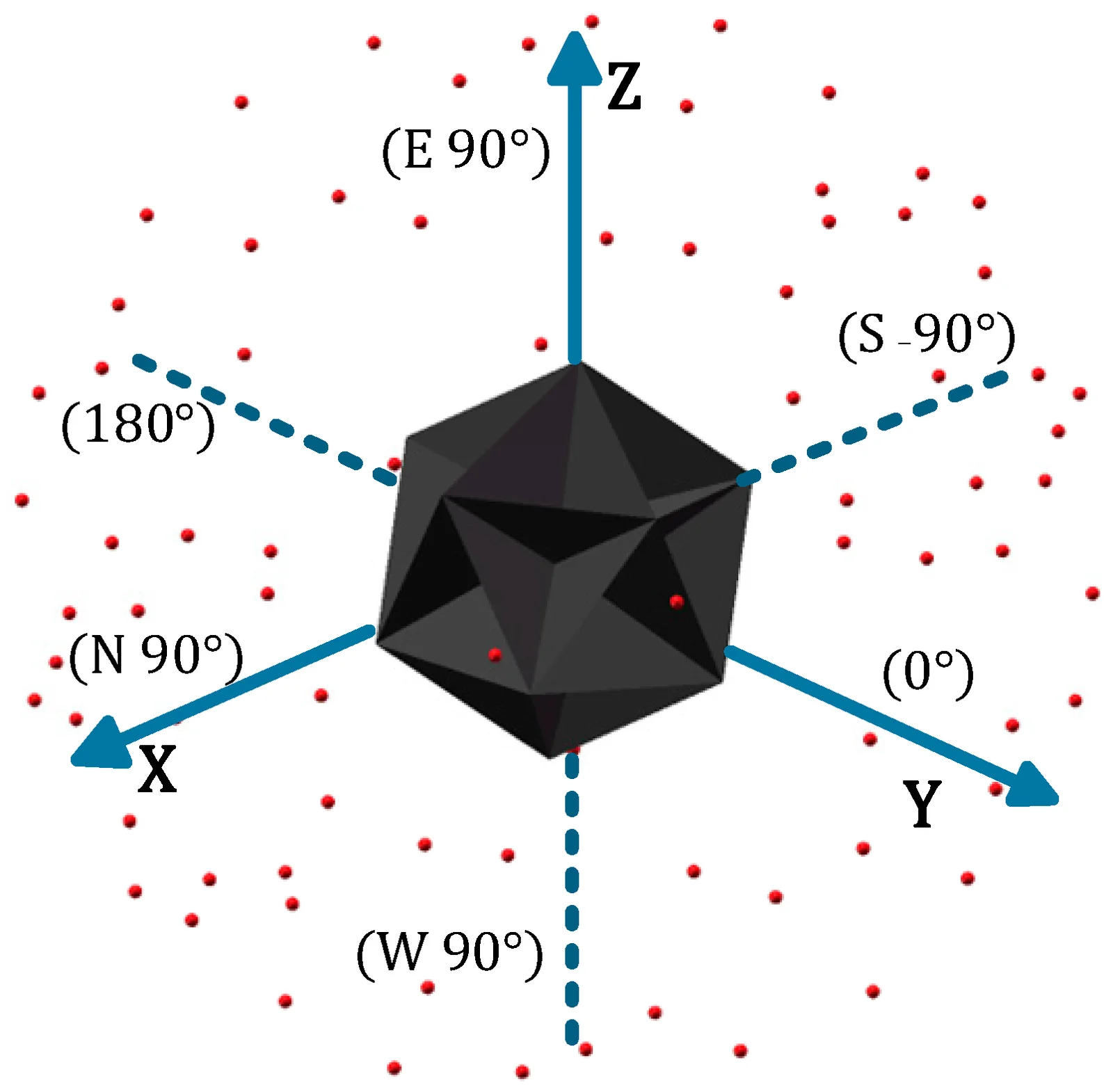
Schematic diagram of the 82-viewpoint omnidirectional observation network.

A geometric model is established using commercial CAD software (SolidWorks 2024), and an omnidirectional observation network consisting of 82 viewpoints is constructed, as shown in Figure 7. To conveniently represent the spatial coordinates and viewing angles of each observation point, a three-dimensional coordinate system is defined with the geometric center of the regular icosahedral corner reflector (or regular icosahedron) as the origin. Drawing on the geographic concepts of longitude and latitude, a spherical longitude–latitude coordinate system suitable for this experiment is introduced. The XOY plane of the three-dimensional coordinate system is taken as the prime meridian plane, and the YOZ plane as the equatorial plane. Ten observation points are arranged on each meridian and each latitude. Since the ten detection points at 90° N and 90° S coincide, the actual total number of observation points is 82.

Based on the above geometric models, CFD software (ANSYS Fluent 2022) is used to generate meshes for both the models and the far field, and to calculate the temperature field. The computed temperature-field results are then imported into dedicated infrared characteristic simulation software, into which the multi-viewpoint observation network is also incorporated. Taking the surface emissivity, temperature, and physical size of the corner reflector (or regular icosahedron) as variables, four groups of experiments are designed, and the initial settings of the parameters for each group are listed in Table 1. According to Planck’s law, the peak radiation wavelength of a target in the temperature range of 60 °C to 80 °C falls within the long-wave infrared region. Therefore, the long-wave infrared band (8–12 μm) is selected as the observation band in this study. In addition, since the distances between the detection points and the target surface are nearly identical in all experimental groups, the attenuation of infrared radiation under the same atmospheric conditions (i.e., atmospheric transmittance) is also approximately the same. It can therefore be treated as an irrelevant variable and omitted from the radiation characteristic modeling, so as to simplify the simulation process.

Based on the given emissivity of the corner-reflector surface material, the first-order and second-order approximate values of the effective emissivity of each cavity aperture are obtained through theoretical calculation, as shown in Table 2. To comprehensively validate the effective emissivity calculation method derived above, six simulation calculations are carried out for each experimental group. Specifically, these include: the RMCM simulation of the corner reflector using the preset emissivity at each viewpoint in the omnidirectional observation network; the theoretical calculation of the spectral radiation intensity of the equivalent regular icosahedron using the second-order and first-order approximate values of the effective emissivity; the RMCM simulation of the equivalent regular icosahedron assigned with the second-order and first-order approximate values of the effective emissivity; and the RMCM simulation of the regular icosahedron assigned with the preset emissivity. By varying the target parameters across the four experimental groups, multiple sets of data are obtained for horizontal comparison, and a quantitative analysis of experimental errors is completed.

### 3.3. Comparative Analysis of Simulation Calculation Results

In the four experimental groups, for the equivalent icosahedron with emissivity assigned as the second-order approximation, the average differences between the results obtained by the theoretical calculation method and the RMCM simulation method are 0.0079 W/(sr∙μm), 0.0080 W/(sr∙μm), 0.0072 W/(sr∙μm), and 0.0175 W/(sr∙μm), respectively. The corresponding ratios to the average values of the RMCM simulation results are 0.28‰, 0.22‰, 0.28‰, and 0.16‰, respectively. For the equivalent icosahedron with emissivity assigned as the first-order approximation, the differences between the results obtained by the two methods are 0.0039 W/(sr∙μm), 0.0096 W/(sr∙μm), 0.0082 W/(sr∙μm), and 0.0167 W/(sr∙μm), respectively, with ratios to the average simulation results of 0.14‰, 0.26‰, 0.31‰, and 0.15‰, respectively. These results further demonstrate the feasibility and reliability of using the RMCM to validate the theoretical approach.

This study takes the 10.4 μm band as an example for result analysis. Although the current simulation experiments do not incorporate atmospheric effects, this band lies in the core region of the long-wave infrared atmospheric window, where atmospheric transmittance is high and water vapor absorption is minimal. Therefore, the conclusions obtained can provide a reliable reference for subsequent extended studies. According to the simulation results at different viewing angles in the multi-view observation network, the results of the four experimental groups are summarized in Table 3.

**Figure 8:**
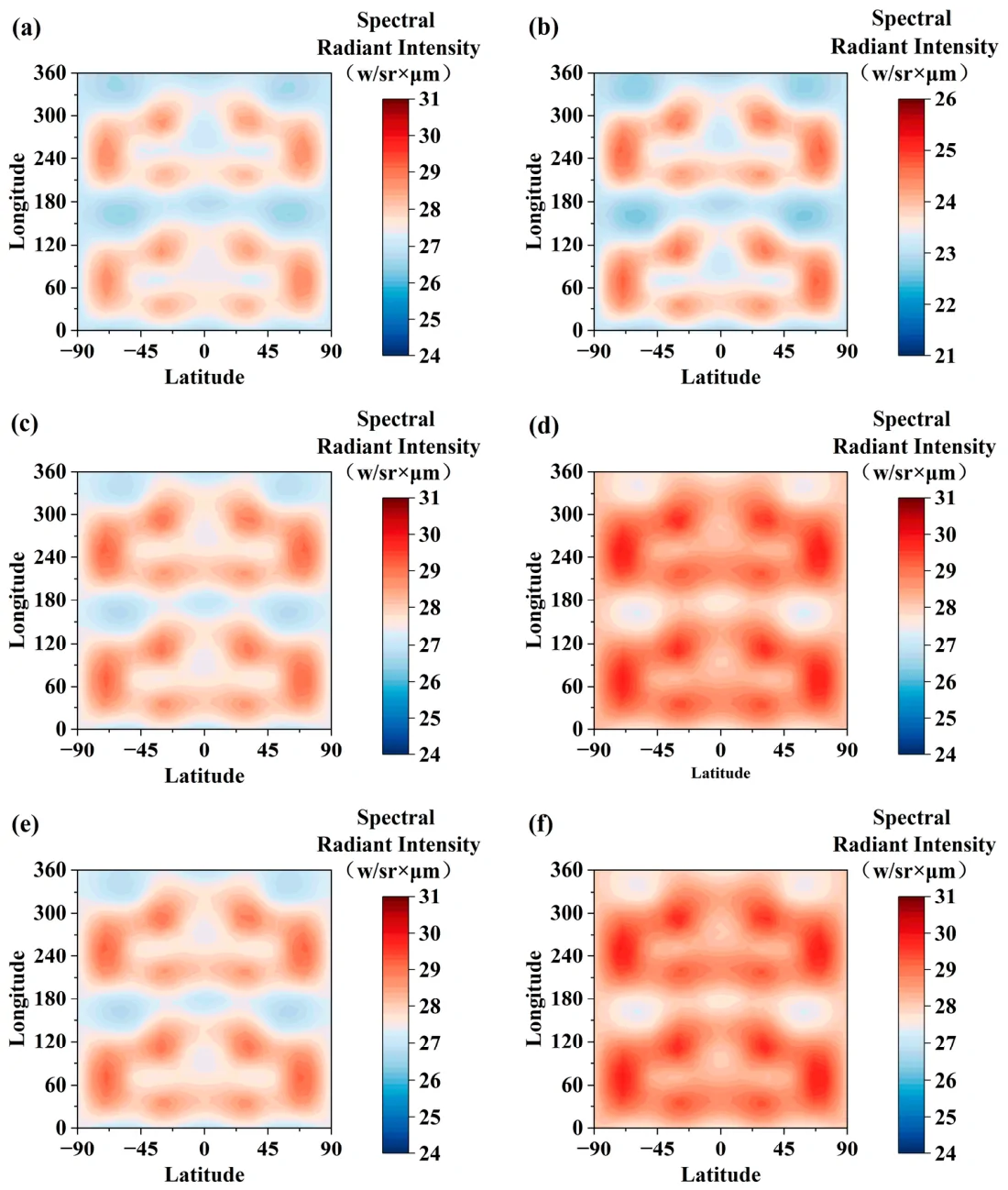
Results of the first experimental group: (**a**) RMCM simulation result of the corner reflector; (**b**) RMCM simulation result of the icosahedron with preset emissivity; (**c**) RMCM simulation result of the icosahedron with second-order approximated emissivity; (**d**) RMCM simulation result of the icosahedron with first-order approximated emissivity; (**e**) theoretical calculation result of the icosahedron with second-order approximated emissivity; (**f**) theoretical calculation result of the icosahedron with first-order approximated emissivity.

**Figure 9:**
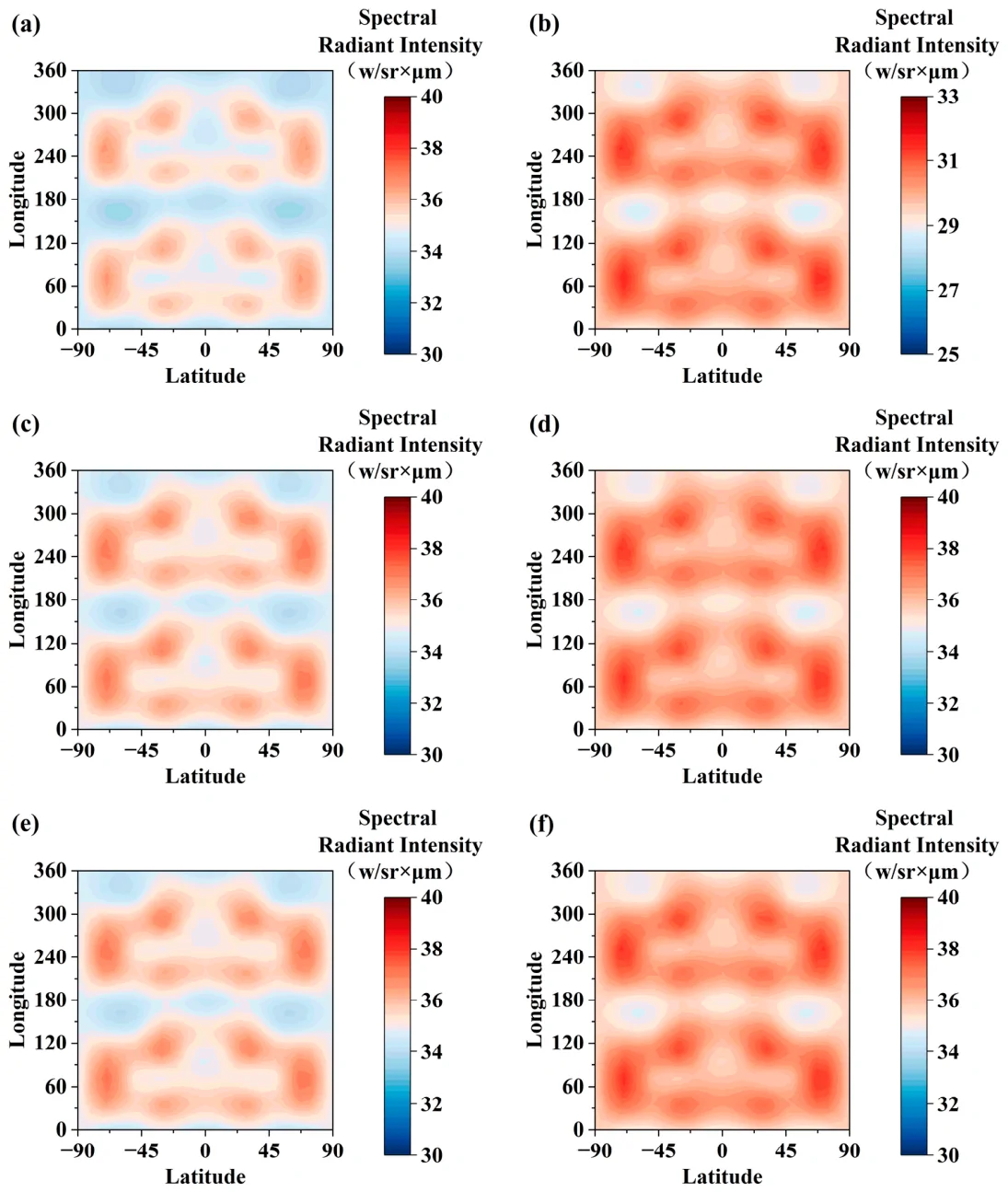
Results of the second experimental group: (**a**) RMCM simulation result of the corner reflector; (**b**) RMCM simulation result of the icosahedron with preset emissivity; (**c**) RMCM simulation result of the icosahedron with second-order approximated emissivity; (**d**) RMCM simulation result of the icosahedron with first-order approximated emissivity; (**e**) theoretical calculation result of the icosahedron with second-order approximated emissivity; (**f**) theoretical calculation result of the icosahedron with first-order approximated emissivity.

**Figure 10:**
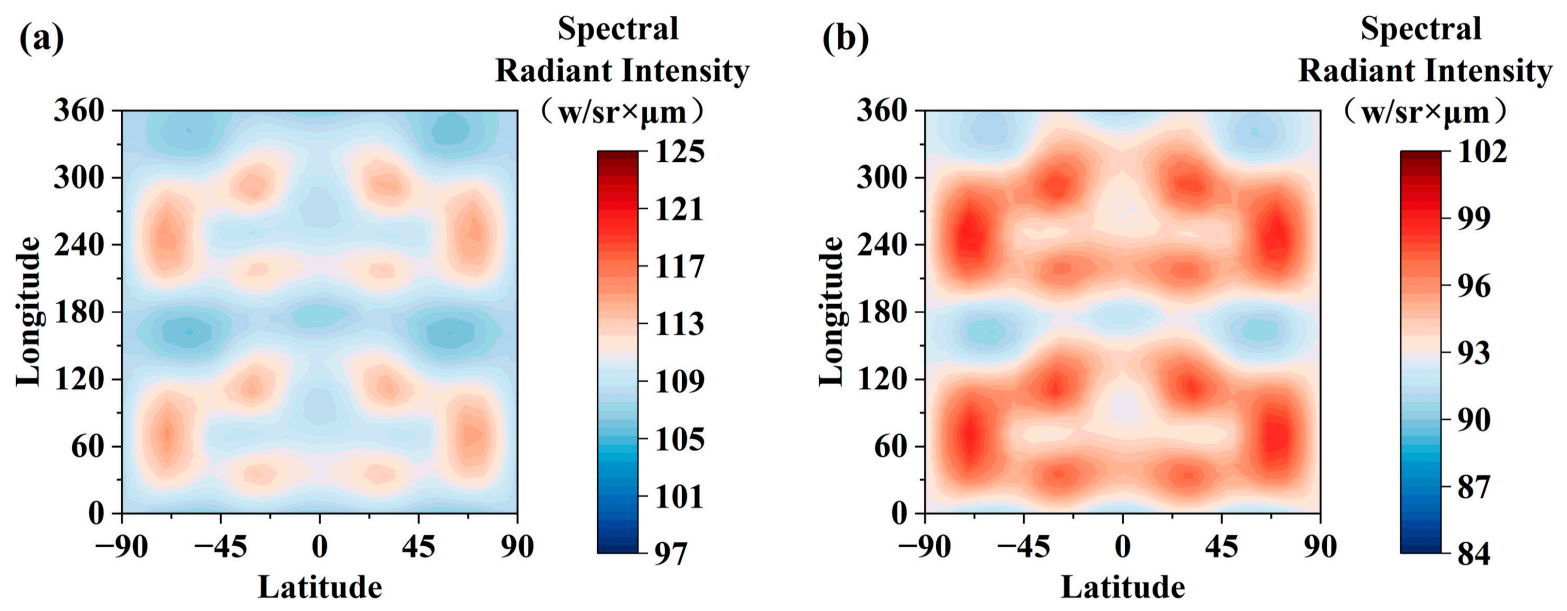

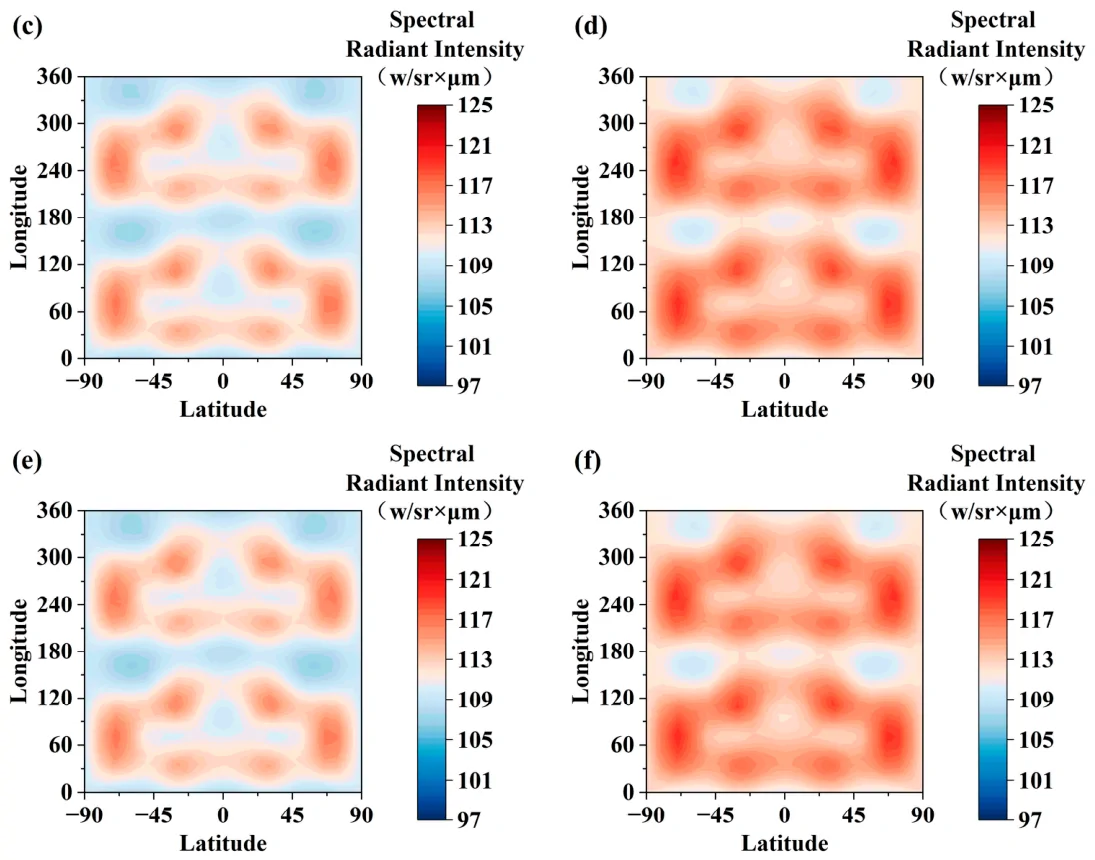
Results of the third experimental group: (**a**) RMCM simulation result of the corner reflector; (**b**) RMCM simulation result of the icosahedron with preset emissivity; (**c**) RMCM simulation result of the icosahedron with second-order approximated emissivity; (**d**) RMCM simulation result of the icosahedron with first-order approximated emissivity; (**e**) theoretical calculation result of the icosahedron with second-order approximated emissivity; (**f**) theoretical calculation result of the icosahedron with first-order approximated emissivity.

**Figure 11:**
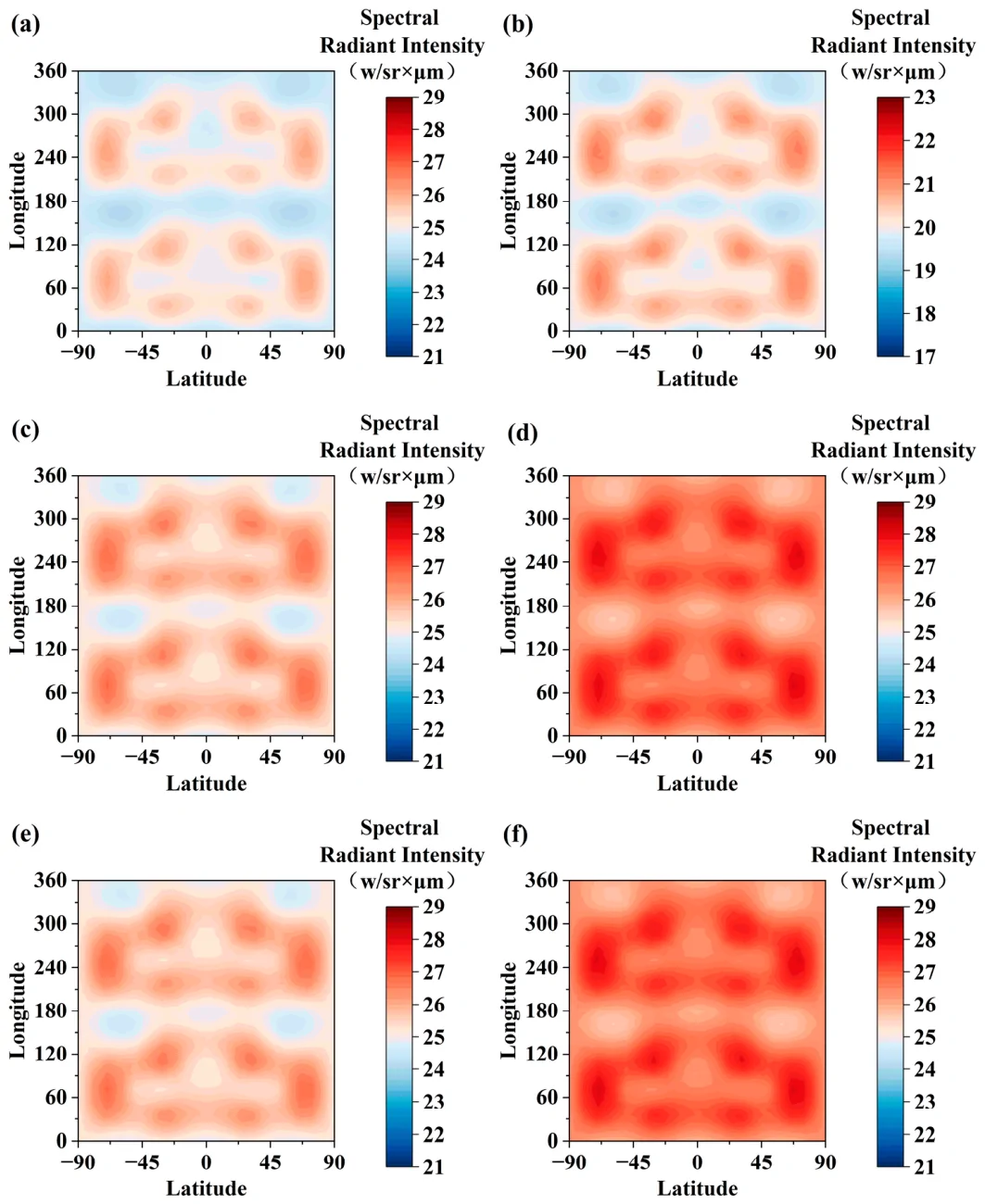
Results of the fourth experimental group: (**a**) RMCM simulation result of the corner reflector; (**b**) RMCM simulation result of the icosahedron with preset emissivity; (**c**) RMCM simulation result of the icosahedron with second-order approximated emissivity; (**d**) RMCM simulation result of the icosahedron with first-order approximated emissivity; (**e**) theoretical calculation result of the icosahedron with second-order approximated emissivity; (**f**) theoretical calculation result of the icosahedron with first-order approximated emissivity.

According to the simulation results at each viewing angle, contour maps of the spectral radiance at 10.4 μm for different target types were generated using a linear interpolation method, so as to present the spatial distribution of target radiance in an overall manner, as shown in Figure 8, Figure 9, Figure 10 and Figure 11. Since the equivalent regular icosahedron and the corner reflector share the same external geometric contour, their infrared radiation characteristics exhibit the same viewing-angle dependence across all four experimental groups. Under the condition of identical target temperature and emissivity, the spectral radiance is proportional to the projected area of the target at the corresponding viewing angle.

In the first experimental group, the mean spectral radiance of the corner reflector in the 10.4 μm band was 27.549 W/(sr·μm). According to Table 3, in the second-order approximation equivalence experiment, the differences between the theoretical value and the RMCM simulation value of the mean spectral radiance of the icosahedron and the mean radiance of the corner reflector were 0.317 W/(sr·μm) and 0.305 W/(sr·μm), accounting for 1.15% and 1.11%, respectively. By contrast, in the first-order approximation equivalence experiment, the corresponding proportions of the two differences were 3.57% and 3.56%, respectively. These results indicate that the error of the equivalent substitution experiment based on first-order approximated effective emissivity is slightly higher than that under the second-order approximated emissivity condition, which is consistent with the basic principle of De Vos theory. Specifically, the derivation of the first-order approximation of effective emissivity considers only the direct escape of radiation, whereas the second-order approximation additionally incorporates the escape of radiation from the cavity opening after reflection from the cavity wall, thus providing a description of radiative transfer within the cavity that is closer to the actual physical process.

In the regular icosahedron control group, where the emissivity was set equal to that of the corner reflector, the mean spectral radiance was 23.602 W/(sr·μm). The difference from the corner reflector was 3.947 W/(sr·μm), corresponding to 14.32% of the mean spectral radiance of the corner reflector, which is far greater than the errors obtained when the icosahedron adopted first-order and second-order approximated effective emissivities. Therefore, under the conditions that the emissivity of the corner reflector was 0.7 and the temperature was 60 °C, the theoretical calculation model of effective emissivity and the equivalent calculation method for the radiative characteristics of the corner reflector are valid.

In the second experimental group, the target temperature was increased to 80 °C, and the mean spectral radiance of the corner reflector at 10.4 μm was 35.010 W/(sr·μm). For the equivalent regular icosahedron with second-order approximated emissivity, the theoretical and simulated mean spectral radiance values were 35.403 W/(sr·μm) and 35.396 W/(sr·μm), respectively. The differences from the mean radiance of the corner reflector were 0.393 W/(sr·μm) and 0.386 W/(sr·μm), accounting for 1.12% and 1.10% of the corner reflector’s mean radiance, respectively. Under the first-order approximated emissivity condition, the theoretical and simulated values were 36.251 W/(sr·μm) and 36.239 W/(sr·μm), respectively, with mean errors relative to the corner reflector of 1.240 W/(sr·μm) and 1.228 W/(sr·μm), corresponding to 3.54% and 3.51% of the corner reflector’s mean radiance. By contrast, the mean spectral radiance of the icosahedron based on the preset emissivity was 29.981 W/(sr·μm), and the corresponding error relative to the corner reflector’s mean radiance reached 14.37%.

These results show that after the target temperature increased, the errors of the equivalent substitution experiments based on first-order and second-order approximated emissivities remained essentially the same as those in the first experimental group. Moreover, under the condition of identical emissivity, the mean radiance of the corner reflector was still significantly higher than that of the regular icosahedron. Assuming that the surface emissivity of the material is temperature-independent, the model error remains essentially stable over the investigated temperature range. This is because the effective emissivity is governed primarily by the cavity geometry and the radiative properties of the material. An increase in temperature changes the radiation intensity according to Planck’s law but does not affect the relative error of the model. Therefore, the theoretical calculation model and the equivalent calculation method for the radiative characteristics of the corner reflector are not dependent on target temperature.

In the third experimental group, the target size was increased: the outer-edge length of the corner reflector was enlarged from 1 m to 2 m, and the equivalent icosahedron model was adjusted accordingly, while all other parameters were kept the same as those in the first experimental group. Under this condition, the mean spectral radiance of the corner reflector was 110.055 W/(sr·μm). For the equivalent icosahedron with second-order approximated emissivity, the theoretical mean spectral radiance was 111.463 W/(sr·μm), and the RMCM simulated value was 111.476 W/(sr·μm), with differences accounting for 1.28% and 1.29% of the mean radiance of the corner reflector, respectively. In the first-order approximation equivalence experiment, the theoretical and RMCM simulated mean spectral radiance values of the icosahedron were 114.129 W/(sr·μm) and 114.111 W/(sr·μm), respectively, and the corresponding differences accounted for 3.70% and 3.69% of the mean radiance of the corner reflector. In the icosahedron control group with emissivity set to 0.7, the mean spectral radiance was 94.414 W/(sr·μm), and the difference amounted to 14.21% of the corner reflector’s mean value.

From a physical perspective, the absence of a significant increase in error with cavity size can be attributed to the scale invariance of the effective emissivity. As a dimensionless quantity, the effective emissivity depends on the cavity geometry rather than its absolute dimensions, provided that the radiative properties of the material remain unchanged and geometric similarity is preserved. When the edge length is increased from 1 m to 2 m, all linear dimensions of the regular triangular-pyramidal cavity are scaled by the same factor. However, the solid angle subtended by the aperture at a surface element on the cavity wall is determined solely by the relative geometry and spatial angles and is therefore invariant under uniform scaling. Consequently, both the first- and second-order approximations of the effective emissivity remain unchanged. Meanwhile, under identical radiance and observation conditions, the detected radiant power is proportional to the projected area of the target. Thus, as the cavity size changes, the radiant powers predicted by the equivalent model and the original structure scale by the same factor, leaving their relative error essentially unchanged.

It can be seen that when the volumes of both the regular triangular pyramid cavity and the corner reflector were increased by a factor of four, the theoretical calculation model and the equivalent calculation method for the radiative characteristics of the corner reflector remained valid and reliable. Therefore, the above method demonstrates a certain degree of robustness with respect to changes in model size.

In the fourth experimental group, the preset emissivity of the corner reflector was changed to 0.6, while all other experimental conditions remained the same as those in the first group. Simulation results showed that the mean spectral radiance of the corner reflector was 25.048 W/(sr·μm). For the equivalent icosahedron with second-order approximated emissivity, the theoretical mean spectral radiance was 25.617 W/(sr·μm), and the RMCM simulated value was 25.611 W/(sr·μm), with differences accounting for 2.27% and 2.21% of the mean radiance of the corner reflector, respectively. For the equivalent icosahedron with first-order approximated emissivity, the theoretical and RMCM simulated mean spectral radiance values were 26.802 W/(sr·μm) and 26.792 W/(sr·μm), respectively, and the corresponding differences accounted for 7.01% and 6.96% of the mean radiance of the corner reflector. In the icosahedron control group with emissivity set to 0.6, the mean spectral radiance was 20.222 W/(sr·μm), and the difference amounted to 19.24% of the corner reflector’s mean value.

It can be seen that after the emissivity was reduced by 0.1, the errors of the equivalent icosahedron experiments, including both first-order and second-order approximations, increased by approximately twofold compared with the other three experimental groups. This is mainly because, in the calculation of the approximated effective emissivity based on De Vos theory, only the radiation emitted by the cavity wall itself and the radiation escaping from the cavity opening after one internal reflection are considered, while the contributions from second and higher-order internal reflections are neglected. When the cavity surface reflectivity is relatively high, the absorption of infrared radiation by the cavity wall is weakened, and the proportion of radiative flux escaping after multiple wall reflections increases significantly. Neglecting the contribution of this multiply reflected escaping radiation leads to an effective emissivity larger than the true value. According to the above experimental results, when the emissivity was 0.7 and 0.6, the errors of the second-order approximation equivalence experiment were only 1.15% and 2.27%, respectively, indicating that this method has a certain degree of reliability when the target emissivity is relatively high. However, its robustness with respect to variations in the target’s intrinsic emissivity still requires further verification.

**Figure 12:**
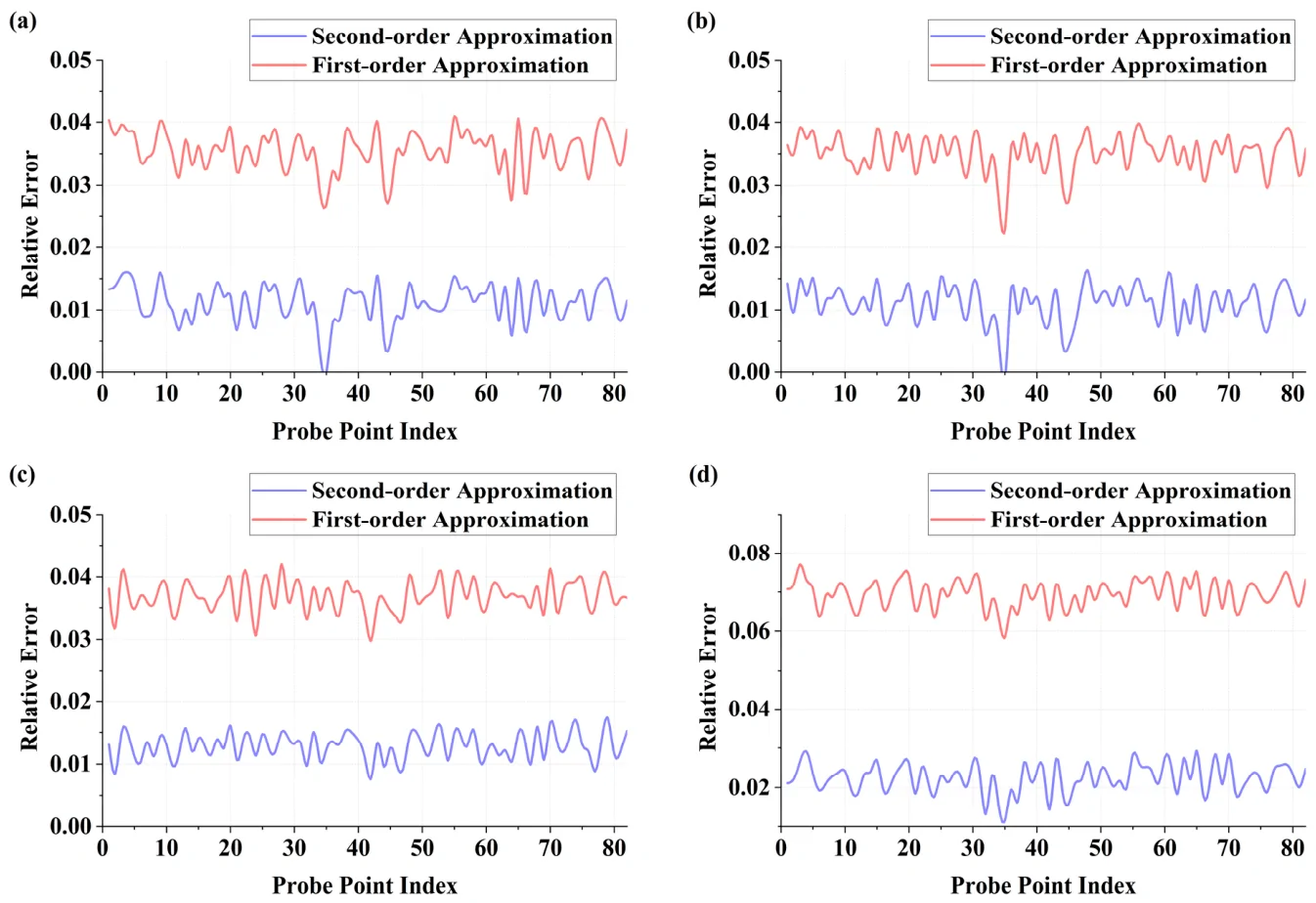
Distribution of relative errors as a function of observation angle. (**a**) Experiment 1; (**b**) Experiment 2; (**c**) Experiment 3; (**d**) Experiment 4.

To provide a more comprehensive evaluation of the accuracy of the equivalent model, additional statistical metrics, including the maximum error and standard deviation, were calculated for the first- and second-order approximation results of the four experiments described above. The corresponding results are summarized in Table 4, while Figure 12 illustrates the error distributions as a function of the observation angle.

As shown in the figure, the errors between the simulation results obtained using the first- and second-order equivalent models and those obtained directly using the corner-reflector model are relatively uniform across different observation angles. Moreover, the maximum errors deviate only slightly from the corresponding mean errors. These results not only further confirm the conclusions presented above but also demonstrate that the proposed method is robust to variations in the observation angle. This robustness can be attributed to the multi-cavity angular-error cancelation effect of the icosahedral corner reflector. Because its cavities are uniformly distributed in space, cavity elements exhibiting stronger and weaker radiation are randomly combined and mutually compensated at any observation angle, enabling the equivalent model to maintain stable performance under omnidirectional observation.

### 3.4. Error Analysis and Correction

According to the experimental results presented in Section 3.3, the simplified equivalent method proposed in this study for characterizing the radiative properties of a composite structure comprising multiple regular triangular-pyramidal cavities exhibits high reliability at relatively high target emissivities and good robustness to variations in target temperature and size. However, its stability under varying target emissivities requires further investigation. To clarify the relationship between the algorithm error and the emissivity of the corner reflector, the emissivity range is extended based on Experiment 4. The expanded initial parameter settings are listed in Table 5.

**Figure 13:**
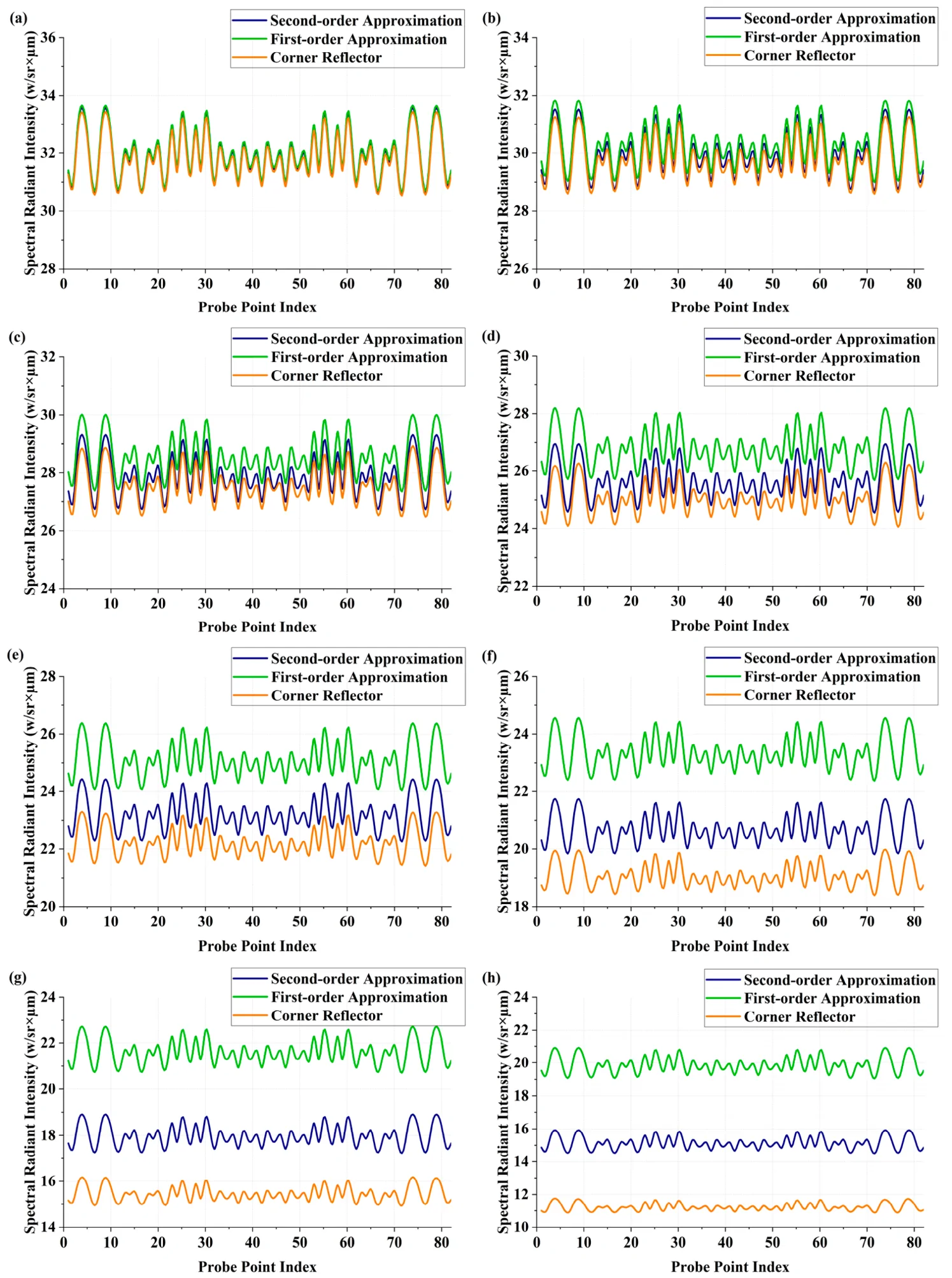
Comparison of expanded experimental results: (**a**) Experiment 4-1; (**b**) Experiment 4-2; (**c**) Experiment 4-3; (**d**) Experiment 4-4; (**e**) Experiment 4-5; (**f**) Experiment 4-6; (**g**) Experiment 4-7; (**h**) Experiment 4-8.

For each experimental group, the RMCM simulation of the corner reflector, the theoretical calculation of the radiative intensity of the equivalent icosahedron based on the first-order approximation, and the theoretical calculation of the radiative intensity of the equivalent icosahedron based on the second-order approximation were carried out using the 82-viewpoint observation network. Among them, Experiments 4-3 and 4-4 directly adopted the corresponding results of Experiment 1 and Experiment 4, respectively. A summary of the experimental results at the 10.4 μm band is presented in Table 6, and the spectral radiative intensity of the target at each observation point is shown in Figure 13.

It can be clearly observed from the experimental data and spline plots that, across the eight experimental groups, the error introduced by the equivalent substitution of the corner reflector increases as its emissivity decreases. Therefore, the reliability of this equivalent substitution method is unsatisfactory for corner reflector targets with relatively low emissivity. The results in Table 6 further indicate that the error of the equivalent approximate solution exhibits an approximately multiplicative growth trend as the surface emissivity of the corner reflector decreases. Specifically, for every 0.1 decrease in emissivity, the second-order approximation error increases by about twofold. In addition, the standard deviation of the experimental error in each group also increases as the emissivity decreases.

The analysis of the error mechanism in Experiment 4 presented in Section 3.3 indicates that, under high-reflectivity experimental conditions, the error arises from the theoretical derivation formula neglecting the multiple-reflection process of radiation within the cavity. Although the error could be compensated by deriving the third-order and higher-order approximations of the effective emissivity, this would require the calculation of multiple solid-angle integrals, thereby greatly increasing the computational complexity. Therefore, this study adopts a correction scheme based on the introduction of an empirical coefficient for error compensation. Specifically, a correction term containing the cube of reflectivity is added to the calculation formula of the second-order approximate effective emissivity, and the correction model is constructed as follows:

(58) $$\varepsilon_{corr}=1-\varepsilon_{1}^{\prime }-\varepsilon_{2}^{\prime }-k\rho^{3}$$

Taking the minimization of the average calculation error over the emissivity range [0.2, 0.9] as the optimization objective, a global traversal search was performed for the coefficient k. Within a reasonable range, candidate values were substituted one by one to evaluate the overall error under each experimental condition. Through comparative screening, it was found that when k=0.23, the errors of all experimental groups remain below 1%, while the average error is also maintained at a relatively low level, as shown in Table 7. In addition to the low mean error, both the maximum error and the standard deviation remain at low levels, demonstrating that the introduction of this empirical coefficient does not compromise the stability of the equivalent model under varying observation angles.

The introduced empirical coefficient term essentially compensates for the higher-order reflection contributions neglected in the original model. After introducing this coefficient, the overall model error is significantly reduced, particularly under low-emissivity conditions. The spectral radiant intensity predicted by the equivalent icosahedral model agrees closely with that obtained using the corner-reflector model, thereby confirming the physical validity of the empirical coefficient correction. Specifically, the second-order approximation accounts for contributions from up to two wall reflections, including the term proportional to $\rho^{2}$. Consequently, the neglected contributions from third- and higher-order reflections are dominated by the $\rho^{3}$ term, providing a physical basis for expressing the correction term as the third power of reflectivity. It should be noted that the coefficient k=0.23 was obtained through global optimization over an emissivity range of 0.2∼0.9 for regular triangular-pyramidal cavities and an icosahedral corner reflector. For cavities with other geometrical configurations, such as general triangular-pyramidal or quadrangular-pyramidal cavities, this coefficient cannot be applied directly because of differences in the corresponding solid-angle integrals. Instead, it must be redetermined through appropriate experiments or numerical calculations.

**Figure 14:**
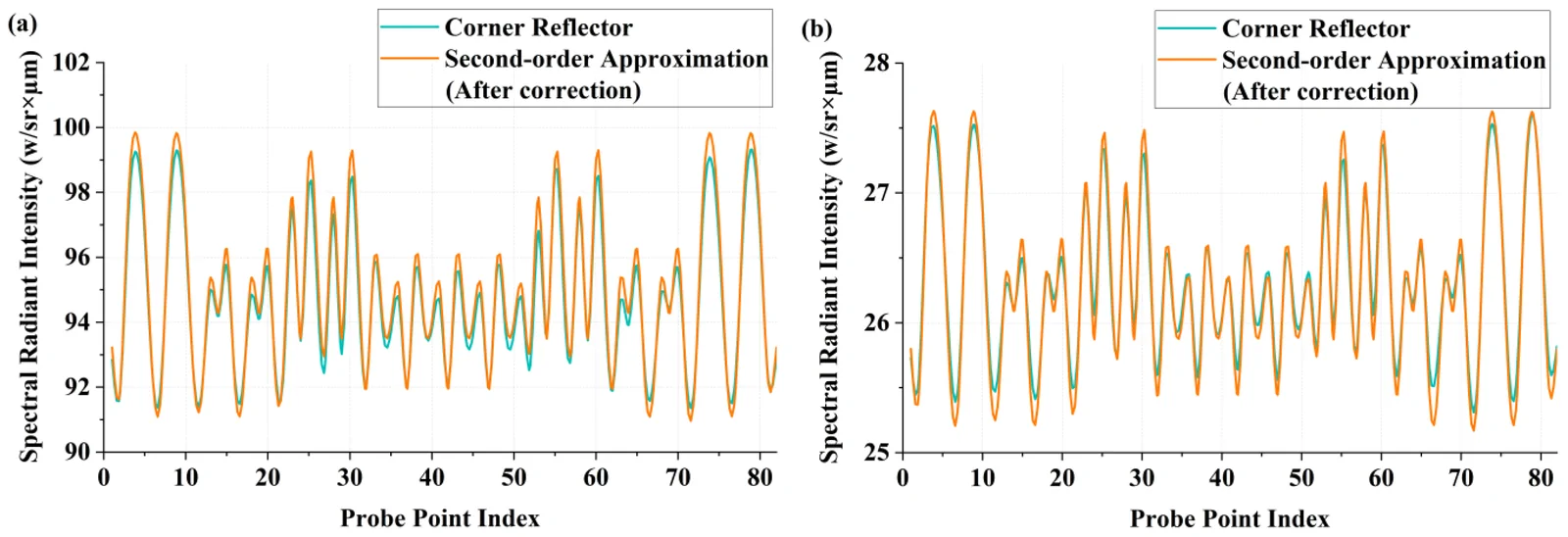
Comparison of validation experimental results: (**a**) validation experiment 1; (**b**) validation experiment 2.

In addition, two further experiments were conducted to verify the reliability and robustness of the proposed approximation method under varying target size and temperature conditions. Based on Experiment 3, the outer edge length of the corner reflector was set to 2 m with an emissivity of 0.55; based on Experiment 2, the temperature of the corner reflector was set to 80 °C with an emissivity of 0.45. The experimental results are shown in Figure 14.

The mean errors in validation experiments 1 and 2 are 0.3% and −0.1%, respectively. In addition, the close agreement between the two curves in the figure indicates a small standard deviation of the error. These results demonstrate that the proposed equivalent substitution method remains highly reliable under variations in target temperature and size. Taken together with the theoretical derivation and simulation-based validation under multiple operating conditions, these results confirm that the corrected effective emissivity model of the cavity and the equivalent substitution method for the corner reflector developed in this study are generally applicable and capable of supporting quantitative infrared radiation calculations for different target temperatures, cavity geometric dimensions, and wall emissivity conditions.

### 3.5. Evaluation of Simulation Computational Efficiency

**Figure 15:**
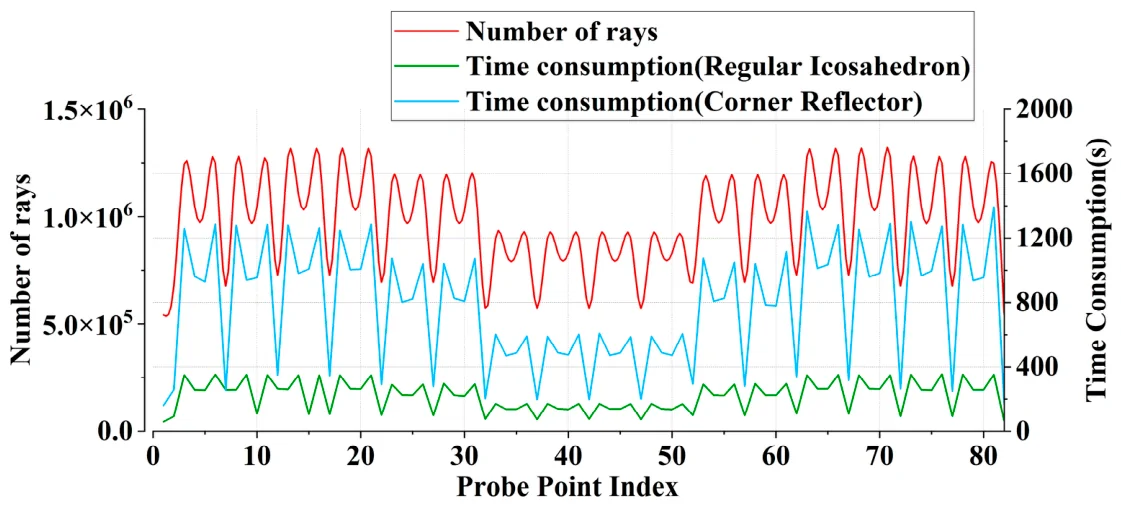
Comparison of Computational Efficiency.

This study carried out simulation experiments on the infrared radiation characteristics of the icosahedral corner reflector and its equivalent regular icosahedron based on the RMCM simulation framework and an improved ray-tracing method. The simulations were performed on a hardware platform equipped with a 12th Gen Intel Core i7-12700 @ 2.10 GHz (12 cores, 20 threads) and 32 GB of RAM. Commercial CFD software was employed for unstructured mesh generation and temperature-field calculations. Because the corner reflector has a complex geometry comprising 20 independent triangular-pyramidal cavities, a refined mesh was required to ensure computational accuracy, resulting in approximately $47.6\times 10^{4}$ cells. By contrast, the equivalent regular icosahedron has regular surfaces and a simple geometry, allowing a converged solution to be obtained with a relatively coarse mesh of approximately $7.5\times 10^{4}$ cells. The temperature-field calculation was considered converged when the residual of the energy equation decreased below $10^{-6}$, the heat fluxes across all walls satisfied the overall energy balance, and the temperature field no longer changed with further iterations. During the RMCM simulation, the ray density for each pixel was set to 10, and the total number of rays was determined by the number of target pixels under each observation angle. Throughout the simulation process, the corner reflector and the regular icosahedron were kept at the same number of pixels under each observation angle. For a single detection point, the average number of pixels was 98,252.88, corresponding to an average of 982,528.8 rays. As a statistical sampling method, the convergence of the RMCM is guaranteed by the law of large numbers. As the number of sampled rays increases, the statistical estimate becomes progressively more stable and eventually converges to its expected value. The simulation time consumed at each detection point is shown in Figure 15.

It can be seen that the computation time of the corner reflector group was significantly higher than that of the regular icosahedron group. Under the condition that the error in the experimental results remained below 1%, the average computation time at each detection point for the corner reflector group was 835.17 s, which is 3.81 times that of the regular icosahedron group (219.41 s). This difference is primarily attributable to the high geometric complexity of the corner-reflector model. Its refined mesh and the multiple radiative reflections within the cavities substantially increase the computational workload. By contrast, the equivalent regular-icosahedron model has significantly lower geometric complexity, enabling rapid convergence with a relatively coarse mesh and eliminating the need to resolve the complex multiple-reflection process within the cavities. System-resource monitoring during the simulations showed that memory usage was closely related to model complexity and mesh size. The peak memory usage was approximately 27.5 GB for the corner-reflector simulation, compared with only 12.2 GB for the equivalent regular-icosahedron model, corresponding to a reduction of approximately 55.6%. These results further demonstrate the significant advantage of the equivalent modeling method in reducing hardware-resource requirements. Overall, the proposed method simultaneously improves computational efficiency and reduces hardware-resource consumption, providing an efficient modeling and computational tool for engineering applications.

### 3.6. Engineering Implementation Method and Uncertainty Analysis of the Results

The equivalent modeling method proposed in this study can be implemented as an engineering computation module for the rapid evaluation of the infrared radiative characteristics of composite structures comprising multiple regular triangular-pyramidal cavities. Once parameters such as the intrinsic material emissivity, target surface temperature, and geometric dimensions have been obtained, the infrared radiative characteristics of the target can be calculated for prescribed detector locations. As shown in Figure 15, the computation time for a single detector location does not exceed 400 s.

For example, in a maritime rescue scenario, calculations can be performed from multiple viewing angles corresponding to those of a rescue aircraft. By entering the prescribed parameters of a heatable corner reflector mounted on a lifeboat, its infrared radiative characteristics in different viewing directions can be obtained. These results can then be used to evaluate its detectability and provide a quantitative basis for subsequent parameter optimization and engineering design. While maintaining computational accuracy, the proposed model substantially improves simulation efficiency and provides a direct quantitative tool for the rapid evaluation of the infrared radiative characteristics of corner reflectors and the design of their heating systems.

In practical engineering applications, however, input parameters such as target emissivity, temperature, and geometric dimensions are inevitably subject to measurement errors or manufacturing tolerances. To evaluate the influence of input-parameter uncertainty on the predictions of the equivalent model, this section quantitatively analyzes the uncertainty in the predicted radiative intensity using an error-propagation method based on the available simulation data. Let the uncertainties in material emissivity, temperature, and edge length be denoted by Δε, ΔT, and ΔL, respectively. Assuming that these input parameters are mutually independent, the combined uncertainty in the predicted infrared radiative intensity, ΔI, can be estimated using the first-order error-propagation formula:

(59) $$∆I\approx \left|\frac{\partial I}{\partial \varepsilon }\right|∆\varepsilon +\left|\frac{\partial I}{\partial T}\right|∆T+\left|\frac{\partial I}{\partial L}\right|∆L$$

Here, ∂I/∂ε, ∂I/∂T, and ∂I/∂L denote the sensitivity coefficients of radiative intensity with respect to emissivity, temperature, and edge length, respectively. These coefficients can be approximated using the central-difference method based on the data obtained from the four sets of experiments described in this section and their extended experiments. Taking Experiment 1 as the baseline, the sensitivity coefficients over different emissivity intervals were estimated using the data presented in Table 7, as summarized in Table 8.

As shown in Table 8, the sensitivity to emissivity increases as the intrinsic emissivity of the material decreases. This is because a reduction in the intrinsic emissivity of each cavity wall of the corner reflector leads to a corresponding increase in its reflectivity, ρ, thereby enhancing the contribution of multiple reflections within the cavities to radiative transfer. Consequently, the effective emissivity becomes more sensitive to variations in the intrinsic material emissivity, resulting in an increased sensitivity of radiative intensity to emissivity.

Differentiating Equation (53) with respect to temperature T yields the temperature sensitivity:

(60) $$\frac{\partial I}{\partial T}=\frac{\varepsilon A}{\pi }\cdot \frac{\partial M_{bb}(\lambda ,T)}{\partial T}$$

At a wavelength of 10.4 μm and over the temperature range of 60~80 °C, substitution into Planck’s law yields temperature-sensitivity values ranging from 0.376 to 0.410, corresponding to a variation of less than 9%. Based on the finite-difference results obtained from Experiments 1 and 2, their mean value of 0.393 is adopted as an engineering estimate of the temperature sensitivity.

Because the radiative intensity is proportional to the square of the edge length, the relative size sensitivity can be defined as follows:

(61) $$\frac{\partial I}{\partial L}=\frac{2I}{L}$$

Therefore:

(62) $$\frac{∆I}{I}=\frac{2∆L}{L}$$

The relative size sensitivity remains constant at 2 and is independent of the specific dimensions. Therefore, the contribution of size uncertainty to the uncertainty in radiative intensity is $2\Delta L/L\cdot I_{0}$. By substituting the engineering measurement uncertainties of emissivity, temperature, and size into Equation (59), the respective contributions of these three parameter uncertainties to the uncertainty in radiative intensity can be calculated. This enables a quantitative analysis of their effects on the infrared characteristics predicted by the model.

## 4. Conclusions

This thesis addresses the problem of low computational efficiency in directly performing high-accuracy numerical simulations of the infrared radiative characteristics of combined multi-triangular-pyramidal cavity structures. Taking a regular icosahedral corner reflector model as the research object, systematic theoretical modeling and simulation validation are carried out based on cavity radiation theory. First, the icosahedral corner reflector is decomposed into twenty regular triangular pyramidal hollow-cavity structures. By using L’Huilier’s formula to calculate the solid angle subtended by the aperture at differential wall elements, first-order and second-order approximate theoretical models for the effective emissivity of the aperture of a regular triangular pyramidal cavity are derived. Second, from the perspective of statistical probability, the cancelation effect of viewing-angle errors among multiple cavities is explained, and an equivalent modeling method based on a “solid regular icosahedron with uniform emissivity” is proposed. This approach simplifies the combined multi-cavity structure into a conventional solid object for solving its infrared radiative characteristics. On this basis, an omnidirectional observation network with 82 viewpoints is established. Using the currently mature RMCM simulation framework and the IRTM [65,66,67], which are widely used in numerical simulations of infrared characteristics, four groups of infrared radiation simulations are carried out in the long-wave infrared band under different target parameter settings. The influences of temperature, size, and emissivity on computational accuracy are quantitatively analyzed. Finally, supplementary experiments are conducted to investigate the error pattern of emissivity on model accuracy. An empirical correction term is introduced to account for the error, thereby constructing a high-accuracy and high-efficiency computational model applicable over the full emissivity range, followed by cross-validation experiments.

The proposed method was systematically evaluated through four sets of controlled experiments, eight sets of extended experiments, and two sets of cross-validation experiments. The results demonstrate that, in terms of computational accuracy, over the emissivity range of 0.2–0.9, the equivalent model achieves a mean error of less than 1% relative to the RMCM simulation results obtained using the original corner-reflector model, with a maximum error not exceeding 2.04%. If the cavity structure is neglected and a solid regular icosahedron is used instead, the radiative intensity is underestimated by more than 14%, confirming the necessity of equivalent modeling. In terms of computational efficiency, the equivalent model requires an average of 219.41 s per detection point, representing a reduction of approximately 73.7% compared with direct simulations using the corner-reflector model. Memory usage is also reduced by approximately 55.6%, indicating simultaneous improvements in computational efficiency and hardware-resource utilization. Regarding robustness, the model error remains stable over a target-temperature range of 60–80 °C, an edge-length range of 1–2 m, and a spectral range of 8–12 μm. The model remains theoretically applicable beyond these ranges. It exhibits strong robustness with respect to temperature and size parameters but is highly sensitive to emissivity, particularly at low emissivities, where an empirical correction term is required to maintain prediction accuracy.

Accordingly, the applicability of the proposed method can be clearly defined as follows. Geometrically, the method is applicable to composite structures formed by regular triangular pyramidal cavities whose apertures have no direct line of sight to one another, such as regular icosahedral corner reflectors and bipyramidal octahedral corner reflectors. In terms of material properties, the applicable intrinsic emissivity range is 0.2–0.9. Regarding operating conditions, the target is assumed to be isothermal and its surface is required to exhibit diffuse-gray characteristics. The method has been validated through simulations in the long-wave infrared band for temperatures of 60–80 °C and edge lengths of 1–2 m. It should be noted that the model remains theoretically applicable when parameters such as temperature, size, and spectral range fall outside the specified ranges; however, its actual performance under such conditions has not yet been validated through simulations. The present method is not directly applicable to targets with more complex geometries or nonuniform temperature distributions. In such cases, additional analytical treatment incorporating a nonuniform temperature-field function, a bidirectional reflectance distribution function (BRDF), or other appropriate models is required.

This study establishes a general infrared radiation modeling paradigm for combined structures composed of multiple regular triangular pyramidal cavities. The technical route presented in this paper—“geometric decomposition, analytical evaluation of effective emissivity, and statistical averaging equivalence”—is not only applicable to icosahedral corner reflectors but can also be directly extended to other combined multi-triangular-pyramidal cavity structures, such as bipyramidal octahedral corner reflectors. The findings of this study have broad prospects for engineering applications. In the fields of infrared stealth and thermal camouflage, the proposed method can provide theoretical support and methodological guidance for the rapid evaluation of the infrared radiation characteristics of complex cavity structures, such as aircraft engine intakes and shipboard heat-dissipation grilles. In defense applications, the method enables quantitative evaluation of the infrared radiative intensity of corner reflectors, thereby supporting the design of dual-mode radar–infrared countermeasure devices. In infrared sensor calibration, the proposed approach can facilitate the design and performance evaluation of standard radiation sources, such as blackbody cavities, while also providing a theoretical basis and simulation tool for the development of dual-mode radar–infrared calibration corner reflectors. In maritime safety applications, the method can further guide the design of heating systems for life-raft-mounted corner reflectors, enabling simultaneous radar and infrared detectability and thereby improving the success rate of maritime search-and-rescue operations.

It should be noted that this study still has certain limitations, all of which arise from necessary simplifications adopted under existing experimental conditions in order to balance computational efficiency, accuracy, and feasibility:

(1) This study assumes that the overall temperature of the combined multi-triangular-pyramidal cavity structure is uniform and does not consider the variation in aperture emissivity of each cavity or in the infrared radiative characteristics of the combined structure under practical conditions where temperature gradients may arise due to nonuniform local heating. Limited by the scope and length of the present work, the main focus of this paper is the equivalent modeling method for the radiative characteristics of combined structures composed of multiple regular triangular pyramidal cavities. In subsequent research, a temperature field distribution function T(x,y,z) will be introduced into the theoretical model to replace the original uniform temperature value, and partitioned integration will be employed to calculate the aperture emissivity and numerically simulate the infrared radiative characteristics of the combined structure under non-isothermal conditions.

(2) In the modeling and theoretical derivation of this study, the surface emissivity of the combined multi-triangular-pyramidal cavity structure is assumed to be uniformly distributed. The possible nonuniform emissivity distribution caused by practical factors such as material aging or local contamination is not taken into account. This simplification is mainly made on the basis of balancing engineering practicality and computational complexity. The experimental results in Table 7 show that when the emissivity deviation is controlled within ±0.1, the resulting error remains below 0.52%, and this level of accuracy is sufficient for the vast majority of practical applications. If an emissivity field distribution function were introduced, the overall computational complexity would increase significantly.

(3) Limited by the length of the thesis, this study investigates the equivalent modeling method for infrared radiative characteristics using only one type of combined multi-regular-triangular-pyramidal cavity structure as an example. However, the derivation method proposed in this paper for the effective emissivity of a regular triangular aperture is applicable to all types of triangular pyramidal cavities, and the equivalent modeling approach can also be extended to combined triangular-pyramidal cavity structures beyond the icosahedral corner reflector. Nevertheless, the higher-order approximation terms (third order and above) differ among different triangular pyramidal cavities. Therefore, the empirical correction coefficient of 0.23 obtained in this study is no longer universally applicable. For targets with low intrinsic emissivity, it is still necessary to re-optimize under the full emissivity range to obtain an appropriate empirical coefficient, or alternatively to further derive higher-order approximation terms for the effective emissivity of the cavity.

(4) The equivalent model developed in this study has thus far been validated only through high-fidelity numerical simulations combining RMCM and IRTM, without comparison against measurements obtained from physical or hardware-in-the-loop experiments. This limitation primarily arises from the engineering challenges associated with fabricating a high-precision regular icosahedral corner reflector and strictly maintaining a uniform temperature across the entire structure. RMCM was selected as the validation benchmark for two main reasons. First, based on the reciprocity principle of radiative transfer, RMCM employs unbiased statistical sampling to account for multiple scattering and complex geometries and is widely used in infrared signature simulation. Second, the IRTM introduced in this study improves the accuracy of intersection determination in conventional ray-path tracing, thereby further reducing systematic deviations caused by floating-point truncation errors and supporting high-fidelity numerical simulation. Consequently, treating the simulation results obtained by combining RMCM with IRTM as the “numerical ground truth” is supported by a rigorous physical and numerical basis. From the perspectives of both the underlying physical mechanisms and the simulation results, the equivalent model achieves a mean error of less than 1% relative to the RMCM results over an intrinsic emissivity range of 0.2–0.9. The remaining error is primarily attributable to the neglect of higher-order reflections at low emissivities. Under actual experimental conditions, if the microscopically rough surface of the corner reflector approximately exhibits diffuse-reflection characteristics and its temperature distribution remains relatively uniform under steady-state thermal conditions, the theoretical assumptions adopted in this study are largely consistent with the physical conditions. It is therefore reasonable to expect that the corrected equivalent model will also maintain a relatively low discrepancy from measured infrared characteristics. The principal sources of deviation may include departures of actual material surfaces from ideal Lambertian behavior, such as a weak specular-reflection component, as well as local temperature gradients within the cavities and reflected contributions from environmental radiation. These practical factors have been addressed in the limitations analysis and will be incorporated into future work by introducing nonuniform temperature fields and more refined bidirectional reflectance distribution function (BRDF) models. Although RMCM is widely used in infrared signature simulation and the IRTM adopted in this study further improves computational accuracy, systematic experimental measurements are still required to confirm the accuracy and robustness of the proposed model under real-world conditions.

Future work will focus on developing infrared radiative modeling and computational methods that more accurately represent practical operating conditions. At the theoretical level, a temperature-field distribution function will be introduced to replace the global isothermal assumption, and piecewise integration will be employed to calculate the effective aperture emissivity under non-isothermal conditions. The proposed methodology will also be extended to composite structures comprising different types of cavities, including quadrangular-pyramidal, pentagonal-pyramidal, and general triangular-pyramidal cavities. At the experimental level, emphasis will be placed on measuring the infrared radiation characteristics of composite structures consisting of multiple regular triangular-pyramidal cavities. High-precision corner-reflector specimens will be fabricated, and an infrared radiation calibration platform with controlled thermal conditions will be established to obtain measured radiative-intensity data. These experimental results will be directly compared with the predictions of the equivalent model proposed in this study, thereby enabling further validation and refinement of the modeling method and improving its applicability to real-world engineering scenarios.

## Figures and Tables

**Table 1:** Simulation parameter settings.

Parameter	Experiment 1	Experiment 2	Experiment 3	Experiment 4
Number of detection points	82			
Detection-point distance (m)	2000			
Infrared band (μm)	8~12			
Number of rays per pixel	10			
Emissivity	0.7	0.7	0.7	0.8
Target temperature (°C)	60	80	60	60
Outer contour edge length (m)	1	1	2	1

**Table 2:** Theoretical calculation values of effective emissivity.

Experiment No.	Material Emissivity	First-Order Approximation	Second-Order Approximation
Experiment 1–3	0.7	0.8461	0.8263
Experiment 4	0.6	0.7947	0.7596

**Table 3:** Summary of Experimental Results at a Wavelength of 10.4 μm.

Parameter	Experiment 1	Experiment 2	Experiment 3	Experiment 4
Mean spectral radiance of the corner reflector (W/(sr∙μm))	27.549	35.011	110.055	25.048
Mean second-order approximation theoretical value (W/(sr∙μm))	27.866	35.404	111.463	25.617
Mean second-order approximation simulation value (W/(sr∙μm))	27.854	35.369	111.476	25.611
Mean first-order approximation theoretical value (W/(sr∙μm))	28.532	36.251	114.129	26.802
Mean first-order approximation simulation value (W/(sr∙μm))	28.528	36.239	114.111	26.792
Mean simulation value of the icosahedron control group (W/(sr∙μm))	23.602	29.981	94.414	20.222

**Table 4:** Error statistics for the four sets of comparative experiments.

Experiment No.	Experiment Group	Mean Error	Maximum Error	Standard Deviation
Experiment 1	Second-Order Approximation	1.11%	1.67%	0.0031
	First-Order Approximation	3.56%	4.24%	0.0031
Experiment 2	Second-Order Approximation	1.10%	1.63%	0.0031
	First-Order Approximation	3.51%	4.09%	0.0030
Experiment 3	Second-Order Approximation	1.29%	1.80%	0.0021
	First-Order Approximation	3.69%	4.30%	0.0025
Experiment 4	Second-Order Approximation	2.21%	3.03%	0.0037
	First-Order Approximation	6.96%	7.90%	0.0036

**Table 5:** Expanded Experimental Parameter Settings.

Experiment No.	Material Emissivity	First-Order Approximation	Second-Order Approximation
Experiment 4-1	0.9	0.9487	0.9465
Experiment 4-2	0.8	0.8947	0.8886
Experiment 4-3	0.7	0.8461	0.8263
Experiment 4-4	0.6	0.7947	0.7596
Experiment 4-5	0.5	0.7434	0.6885
Experiment 4-6	0.4	0.6921	0.6131
Experiment 4-7	0.3	0.6408	0.5332
Experiment 4-8	0.2	0.5895	0.4489

**Table 6:** Summary of Expanded Experimental Results.

Experiment No.	Mean Spectral Radiance of the Corner Reflector (W/(sr∙μm))	Mean Second-Order Approximation Theoretical Value (W/(sr∙μm))	Mean First-Order Approximation Theoretical Value (W/(sr∙μm))	Second-Order Error	Second-Order SD	First-Order Error	First-Order SD
Experiment 4-1	31.824	31.919	31.993	0.30%	0.0012	0.53%	0.0012
Experiment 4-2	29.768	29.967	30.263	0.67%	0.0015	1.66%	0.0015
Experiment 4-3	27.549	27.866	28.532	1.15%	0.0031	3.57%	0.0031
Experiment 4-4	25.048	25.617	26.802	2.27%	0.0037	7.01%	0.0036
Experiment 4-5	22.225	23.220	25.071	4.48%	0.0035	12.8%	0.0038
Experiment 4-6	19.065	20.675	23.341	8.44%	0.0051	22.42%	0.0057
Experiment 4-7	15.454	17.981	21.610	16.35%	0.0061	39.84%	0.0073
Experiment 4-8	11.234	15.139	19.879	34.76%	0.0088	76.95%	0.0116

**Table 7:** Summary of experimental results after error correction.

Experiment No.	Mean Spectral Radiance of the Corner Reflector (W/(sr∙μm))	Corrected Approximate Value of Effective Emissivity	Corrected Mean Spectral Radiation Intensity of the Icosahedron (W/(sr∙μm))	Mean Error	Maximum Error	Standard Deviation
Experiment 4-1	31.824	0.9463	31.91162	0.27%	0.59%	0.0012
Experiment 4-2	29.768	0.8868	29.90459	0.46%	0.84%	0.0015
Experiment 4-3	27.549	0.8201	27.65636	0.39%	0.93%	0.0031
Experiment 4-4	25.048	0.7449	25.12039	0.29%	0.91%	0.0034
Experiment 4-5	22.225	0.6598	22.25015	0.12%	0.74%	0.0034
Experiment 4-6	19.065	0.5634	18.9991	−0.35%	1.37%	0.0047
Experiment 4-7	15.454	0.4543	15.3207	−0.87%	2.04%	0.0051
Experiment 4-8	11.234	0.3311	11.16841	−0.59%	1.82%	0.0064

**Table 8:** Sensitivity Coefficients of Radiative Intensity over Different Emissivity Intervals.

Emissivity Interval	Difference in I	Sensitivity Coefficient
0.9~0.8	2.01	20.07
0.8~0.7	2.25	22.48
0.7~0.6	2.54	25.36
0.6~0.5	2.87	28.70
0.5~0.4	3.25	32.51
0.4~0.3	3.68	36.78
0.3~0.2	4.15	41.52

## Data Availability

The original contributions presented in this study are included in the article. Further inquiries can be directed to the corresponding author.
